# Jasmonic acid regulates lignin deposition in poplar through JAZ5-MYB/NAC interaction

**DOI:** 10.3389/fpls.2023.1232880

**Published:** 2023-07-21

**Authors:** Xin Zhao, Xuemei Jiang, Zeyu Li, Qin Song, Changzhen Xu, Keming Luo

**Affiliations:** ^1^Chongqing Key Laboratory of Plant Resource Conservation and Germplasm Innovation, Integrative Science Center of Germplasm Creation in Western China (Chongqing) Science City, School of Life Sciences, Southwest University, Chongqing, China; ^2^Lab of Plant Cell Engineering, Southwest University of Science and Technology, Mianyang, Sichuan, China; ^3^Key Laboratory of Eco-environments of Three Gorges Reservoir Region, Ministry of Education, School of Life Sciences, Southwest University, Chongqing, China

**Keywords:** JAZ protein, secondary cell wall, lignin deposition, poplar, jasmonic acid

## Abstract

Jasmonic acid (JA) is a phytohormone involved in plant defense, growth, and development, etc. However, the regulatory mechanisms underlying JA-mediated lignin deposition and secondary cell wall (SCW) formation remain poorly understood. In this study, we found that JA can inhibit lignin deposition and SCW thickening in poplar trees through exogenous MeJA treatment and observation of the phenotypes of a JA synthesis mutant, *opdat1*. Hence, we identified a JA signal inhibitor PtoJAZ5, belonging to the TIFY gene family, which is involved in the regulation of secondary vascular development of *Populus tomentosa*. RT-qPCR and GUS staining revealed that *PtoJAZ5* was highly expressed in poplar stems, particularly in developing xylem. Overexpression of *PtoJAZ5* inhibited SCW thickening and down-regulated the expression of SCW biosynthesis-related genes. Further biochemical analysis showed that PtoJAZ5 interacted with multiple SCW switches NAC/MYB transcription factors, including MYB3 and WND6A, through yeast two-hybrid and bimolecular fluorescent complementation experiments. Transcriptional activation assays demonstrated that MYB3-PtoJAZ5 and WND6A-PtoJAZ5 complexes regulated the expression of lignin synthetic genes. Our results suggest that PtoJAZ5 plays a negative role in JA-induced lignin deposition and SCW thickening in poplar and provide new insights into the molecular mechanisms underlying JA-mediated regulation of SCW formation.

## Introduction

The secondary cell wall (SCW) is a crucial component of wood, composed of lignin, cellulose, and hemicelluloses. It provides mechanical support for water transport and physical defense against pathogenic bacteria in woody plants. Lignin, a phenolic polymer, exists in a highly crosslinked state in the SCW of plants and is one of the most important substances for plants to resist pathogenic microorganisms and abiotic stresses (Vanholme et al., 2010).

Lignin is composed of three main types of monolignols: p-hydroxyphenyl (H), guaiacyl (G), and syringyl (S) units. The biosynthesis of monolignols occurs through the phenylpropanoid pathway and involves an 11-step enzymatic reaction in the cytoplasm, requiring the participation of various enzymes, including phenylalanine ammonia lyase (PAL), 4-coumarate-CoA ligase (4CL), cinnamate 4-hydroxylase (C4H), cinnamoyl-CoA reductase (CCR), p-hydroxycinnamoyl-CoA: quinate/shikimate p-hydroxycinnamoyltransferase (HCT), cinnamyl alcohol dehydrogenase (CAD), p-coumaroyl shikimate 3-hydroxylase (C3’H), caffeoyl-CoA O-methyltransferase (CCoAOMT), caffeic acid O-methyltransferase (COMT), and ferulate-5-hydroxylase (F5H) (Vanholme et al., 2010). Finally, monolignols are transported across the plasma membrane to the apoplast, where they undergo oxidative polymerization and crosslinking by peroxidases and/or laccases to form the lignin polymer, which stabilizes the cell wall structure (Freudenberg, 1959; Vanholme et al., 2019). Cellulose is synthesized by a cellulose synthase complex (CSC) located in the plasma membrane, first forming β-1,4-D-glucan chains, and then 24 or 36 β-1,4-D-glucan chains are polymerized to form microfibrils, cellulose microfibres provide essential load-bearing properties to cell walls (Mutwil et al., 2008). Xylan is the main component of hemicellulose, it is mainly composed of linear chains of xylose linked by β-1,4 bonds, synthesized in the Golgi apparatus, and then transported to the cell membrane to participate in cell wall synthesis (Rennie and Scheller, 2014).

SCW biosynthesis is a complex and highly regulated process that involves multiple transcription factors. In model plants, such as Arabidopsis (*Arabidopsis thaliana*) and poplar, a multi-stage feedback regulatory network of SCW synthesis has been established, mainly composed of NAC/MYB transcription factors (Mccahill and Hazen, 2019). NAC transcription factors, including VND1-7, NST1, NST2, and NST3/SND1 in Arabidopsis (Kubo et al., 2005; Zhong et al., 2006; Mitsuda et al., 2007), and PtrWNDs in poplar (Zhong et al., 2010), act as primary switches to initiate the transcriptional regulation of SCWs. Additionally, MYB transcription factors, such as PtrMYB02/03/20/21 in poplar (Wilkins et al., 2009; Mccarthy et al., 2010; Chai et al., 2014), act as secondary switches to activate the transcription of SCW synthase genes. The activation of these switch factors is closely related to plant growth and development, and is precisely regulated by plant hormones.

Jasmonic acid (JA) and its derivatives are lipid-derived phytohormones that are widely involved in the regulation of plant growth, development, and defense response (Staswick and Tiryaki, 2004). Recent studies have revealed that JA is also involved in the lignification process of plant cells. For example, exogenous methyl jasmonate (MeJA) treatment enhances lignin deposition and SCW thickening in Arabidopsis (Sehr et al., 2010), and mechanical injury to plants causes a rapid increase in JA content and lignin synthesis (Denness et al., 2011). Additionally, researchers cultured pine seedlings at an angle and found that JA was consistently distributed in the upper part of the angled stems, where G-type lignin was predominantly present. (Salazar et al., 2019). These findings imply that there is a close relationship between JA and lignin synthesis and secondary cell wall thickening, although the molecular mechanisms involved in this relationship remain unclear.

The main signal transduction pathway of JA is mediated by JAZ (JASMONATE-ZIM-DOMAIN) proteins (Chini et al., 2007). In the absence of JA, JAZ proteins bind to transcription factors, such as MYC2 (Chen et al., 2011), inhibiting their transcriptional activity (Thines et al., 2007). When JA level is elevated, the bioactive derivative JA-Ile binds to the receptor COI1 protein, which then interacts with JAZ proteins, promoting their ubiquitination and degradation by the 26S proteasome, and ultimately leading to the activation of downstream gene expression (Katsir et al., 2008; Kramell et al., 2009). Previous research has demonstrated that MeJA treatment and injury can both increase the expression of the genes involved in lignin production (Tianpei et al., 2015; Hyde et al., 2018; Valenzuela-Riffo et al., 2020), but the regulatory network underlying this process remains to be fully elucidated.

In this study, we investigated the role of JA signaling in regulating lignin deposition and SCW thickening in poplar. We employed the *opdat1* mutant, a poplar JA synthesis mutant obtained from our previous study (Zhao et al., 2021), and confirmed that JA deficiency leads to a reduction in secondary cell wall thickness. Treatment of wild-type poplar with exogenous methyl jasmonate (MeJA) induced the expression of lignin synthesis genes. Furthermore, *PtoJAZ5*-overexpression poplar inhibited JA signal transduction, resulting in a block in the biosynthesis of the SCW. We used yeast two-hybrid (Y2H) and bimolecular fluorescence complementation (BiFC) experiments to confirm that PtoJAZ5 interacts with WND and MYB transcription factors involved in secondary wall synthesis. In addition, our transient transactivation assays of GUS activity showed that PtoJAZ5 suppresses transcriptional activation of downstream SCW synthesis genes by WND6A and MYB3. These findings suggest that JA signaling can regulate the transcript levels of lignin and SCW synthesis genes through PtoJAZ proteins, thereby affecting lignin deposition and SCW thickening in poplar.

## Materials and methods

### Plant materials and growth conditions

Poplar plants (*Populus tomentosa* Carr. clone 741) were grown in a greenhouse at 23-25°C, 16 h light/8 h dark, 5000 lux supplementary light, and humidity 60%. The Col-0 seedlings of *Arabidopsis thaliana* were grown in a incubator at 22°C, 16 h light/8h dark, 10,000 lux supplementary light, and humidity 80%. Arabidopsis seeds were germinated on MS medium (8.5 g/L Agar and 30 g/L sucrose) and placed in a lighted incubator. Arabidopsis seedlings were transferred to nutrient soil and continued to be incubated and growth in a light incubator. The culture conditions: 80% humidity, 20–23°CC, 16/8 h light/dark cycle and 555 mmol m−2 s−1 supplemental light (Murashige and Skoog, 1962). Arabidopsis transformation using 30-day-old plants by floral dip method (Zhang et al., 2006). For poplar transformation, the leaf disk method was used to the pCXSN-PtoJAZ5 vector according to the methods previously (Jia et al., 2010a).

Exogenous jasmonic acid (JA) treatment of wild-type (WT) poplars was performed by continuous watering with a 1 mM solution of methyl Jasmonate (MeJA) for one month, and the plants were cultured in a greenhouse and observed for phenotype.

### Cloning of *PtoJAZ5* cDNA and sequence analysis

Total RNA was extracted from poplar stems and reverse transcribed into cDNA using a kit (TAKARA), specific primers were designed and the full-length coding sequence of *PtoJAZ5* (831 bp) was amplified by Polymerase Chain Reaction (PCR). The amplified fragments were then cloned into pCXSN vector (Chen et al., 2009). For construction of the *PtoJAZ5pro::GUS* reporter vector, the 1857-bp promoter fragment was amplified and assembled into the pCAMBIA1305 vector. All plant expression vectors were used to transform plant material using the *Agrobacterium*-mediated leaf disc method according to previous study (Jia et al., 2010b). The software DNAMAN9 (Lynnon Biosoft, San Ramon, CA, USA) was used for multiple sequence alignments, and the software MEGA7.0 was used for phylogenic analysis using the neighbor-joining (NJ) method.

### RT-qPCR analysis

**Figure 1:**
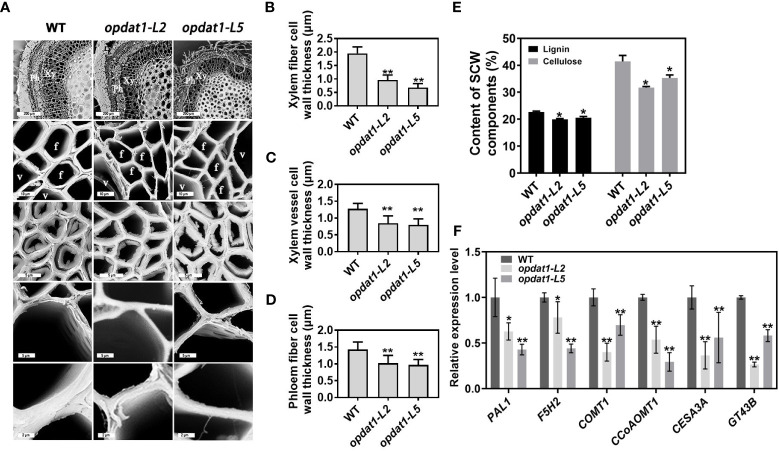
Secondary cell wall thinning in the *opdat11* poplar mutant. **(A)** Scanning electron microscope observation of 5th internode stem section of transgenic poplar, from bottom to top, magnified xylem fiber cells, magnified xylem vessel cells, phloem fiber cells, xylem cells and stem cross-sectional anatomy. The scale bars from bottom to top = 5 μm, 2 μm, 5 μm, 10 μm, 200 μm. Xy, xylem; Ph, phloem; V, vessel cells; F, fiber cells. **(B)** Xylem fiber cell wall thickness statistics. **(C)** Xylem vessel cell wall thickness statistics. **(D)** Phloem fiber cell wall thickness statistics. **(E)** Determination of lignin and cellulose content of three-month-old poplar stems. **(F)** RT-qPCR was performed to detect the expression levels of SCW synthesis genes of three-month-old poplar stems. The error bars indicate the standard deviation of the data from the three biological replicates in the experiment. Asterisks indicate that the data from the experimental replicates show significant differences based on Student’s *t*-test (*P < 0.05; **P < 0.01).

Plant materials such as roots, stems, xylem, bast, old and young leaves in good growth condition were selected and ground thoroughly using liquid nitrogen, and then total RNA was extracted using the Trizol Reagent (Tiangen, China), followed by reverse transcription of cDNA using the PrimeScript™ RT reagent Kit (Takara, Shiga, Japan). Specific quantitative primers were designed as needed, and the quantitative RT-qPCR was performed using SYBR Premix ExTaq™ (Takara) in a TP800 Real-Time PCR machine (Takara). The *UBQ11* gene was used as an internal control in poplar and *AtUBC* in Arabidopsis. The RT-qPCR reaction conditions were 95°C, 30 s; 40 cycles: 95°C, 5 s; 60°C, 1 min; 95°C, 15 s; 60°C, 30 s; 95°C, 15 s. cycles: 95°C, 5 s; 60°C, 1 min; 95°C, 15 s; 60°C, 30 s; 95°C, 15 s. Three biological replicates were required for each gene of the RT-qPCR, and the primers involved were listed in **Table S1**. To analyze their expression levels, the 2^-ΔΔCt^ method (Livak and Schmittgen, 2001) was used in **Figure 1F** and the 2^-ΔCt^ method (Chiba-Falek et al., 2006) was used in other figures.

### Subcellular localization of PtoJAZ5

The full-length coding sequence of *PtoJAZ5* was amplified *via* the specific primer and assembled into the pCAMBIA1300 vector. Then the tobacco leaf flesh epidermal cells were transformed using *A. tumefaciens* mediated transformation method. Empty vector *35S-GFP* was used as positive control. 4’,6-diamidino-2-phenylindole (DAPI) was used to stain the nucleus of cells. A confocal laser microscope (Olympus FV1200, Tokyo, Japan) was used to detected the DAPI and GFP fluorescent signals.

### Yeast two-hybrid assay

Full-length and mutant DNA sequences of *PtoJAZ5* were amplified and constructed into the PGBKT7 (BD) vector, while full-length DNA sequences of *PtoMYC2*, *PtoMYB3/2/20/21/74*, and *PtoWND1A/1B/2A/2B/3A/3B/4A/4B/5A/5B/6A/6B* were amplified and constructed into the PGADT7 (AD) vector. Positive controls included AD-T and BD-53 vectors, while negative controls consisted of AD-T and BD-lam, BD and AD empty vectors. Y2H gold strain was used in yeast experiments, yeast transformation was performed using the PEG/LiAC method according to the Yeast Protocol Handbook (Clontech), and transformed yeast colonies were selected on nutrient-deficient medium. Transcriptional self-activation verification was performed using media lacking histidine, adenine, and tryptophan (SD-Ade-His-Trp) or only lacking tryptophan (SD-Trp), while protein interaction verification was performed using media lacking leucine and tryptophan (SD-Leu-Trp) or lacking tryptophan, leucine, histidine, and adenine (SD-Leu-Trp-Ade-His). Transcription activation activity was identified using X-α-gal, a chromogenic substrate for yeast galactosidase (MEL1). The transformed yeast cells were grown in a 30°C constant temperature incubator for 3-5 days.

### Bimolecular fluorescent complementation assay

The ORF of *PtoJAZ5* was cloned into the PXY106 vector containing the N-terminal of YFP and formed into a complete fusion expression vector. The ORF of *PtoMYB3* and *PtoWND6A* were cloned into the PXY104 vector containing the C-terminal of YFP and formed into a complete fusion expression vector. The resulting constructs were transfered into tobacco leaf flesh epidermal cells using *A. tumefaciens* mediated transformation method. A confocal laser microscope (Olympus FV1200, Tokyo, Japan) was used to detected the DAPI and YFP fluorescent signals after 2-3 days of incubation.

### Scanning electron microscope analysis and histochemical staining

Poplar and Arabidopsis stems were cross-sectioned by hand or semi-automatic slicers (FINESSE 325, Thermo, Runcorn, UK). Histochemical staining including phloroglucinol-HCl, toluidine blue and GUS staining of stem cross sections was performed according to previously described (Li et al., 2015). Scanning electron microscope (Phenomtm Pure FEI, Eindhoven, Netherland) was used to observe the stem cross-sections according to the operation manual.

### Immunohistochemical localization of JA

For immunocytochemical analysis of JAs, stem samples were fixed with 4% (w/v) 1-ethyl-3-(3-dimethyl aminopropyl)-carbodiimide hydrochloride (Merck KgaA) in PBS, embedded in polyethylene glycol (PEG) 1500, and immunostained with an anti-JA antibody as reported for immunocytochemical examination of JAs (Mielke et al., 2011). Refer to the previous description for the detailed operation steps (Fu et al., 2021).

### Determination of the component of SCW

Klason and acid-soluble lignin methods as previously described (Li et al., 2015) were used to measure the total lignin content of poplar stems. Cellulose, pectin and hemicellulose were determined using Peng method (Peng et al., 2000; Fan et al., 2020).

### Statistical analyses

The ImageJ (https://imagej.nih.gov/ij/) (Collins, 2007) software was used for image data measurement, the GraphPad Prism 7 software (Mitteer et al., 2018) was used for data analysis and statistics and One-way ANOVA followed by Dunnett’s test analysis was performed to evaluate statistical significance. For pairwise comparisons, Dunnett’s test was utilized in the statistical analysis.

### Transient expression and GUS activity assay

The promoter sequences of the lignin synthesis genes *PtoCCR2, PtoCOMT2, PtoCCoAOMT1* and *PtoMYB3* were amplified and fused to the *GUS* reporter gene into the PCXGUS-P vector. *Agrobacterium* cells carrying the *35S-PtoJAZ5*, *35S-PtoMYB3* and *35S-PtoWND6A* constructs were used as effectors. Empty vector as negative control. Infiltrating into 2-week-old tobacco leaf flesh epidermal cells with different mixes contain the effectors and reporters of *Agrobacterium* spp. After three days of cultivation, transgenic tobacco leaves were then collected and GUS activity was quantitatively measured by a spectrophotometry according to previously method (Jefferson et al., 1987).

## Results

### Thinning of stem SCW in the poplar *opat1* mutant

To determine the distribution of JA in poplar stem, we conducted immunocytochemical detection experiments and observed that JA is predominantly located in the phloem and developing xylem regions (**Figure S1**), suggesting its potential role in the regulation of xylem development. To further explore the essential role of JA in SCW development, we observed sections of the JA synthesis mutant *opdat1* (Zhao et al., 2021) under scanning electron microscope, which revealed significant reduction in SCW thickness of xylem fiber cells, xylem vessel cells and phloem fibers (**Figures 1A-D**). Furthermore, the lignin and cellulose content of SCW also significantly decreased in *opdat1* mutants (**Figure 1E**), while the xylan content did not differ significantly. RT-qPCR analysis further demonstrated a significant down-regulation in the expression level of key SCW synthesis-related enzyme genes, including *PAL1, F5H2, COMT1, CCoAOMT1, CESA3A*, and *GT43B* in the *opdat1* mutant poplar (**Figure 1F**). These findings suggest that the decreased JA content leads to delayed SCW development in poplar.

### Exogenous MeJA treatment enhanced lignin deposition and SCW thickening

**Figure 2:**
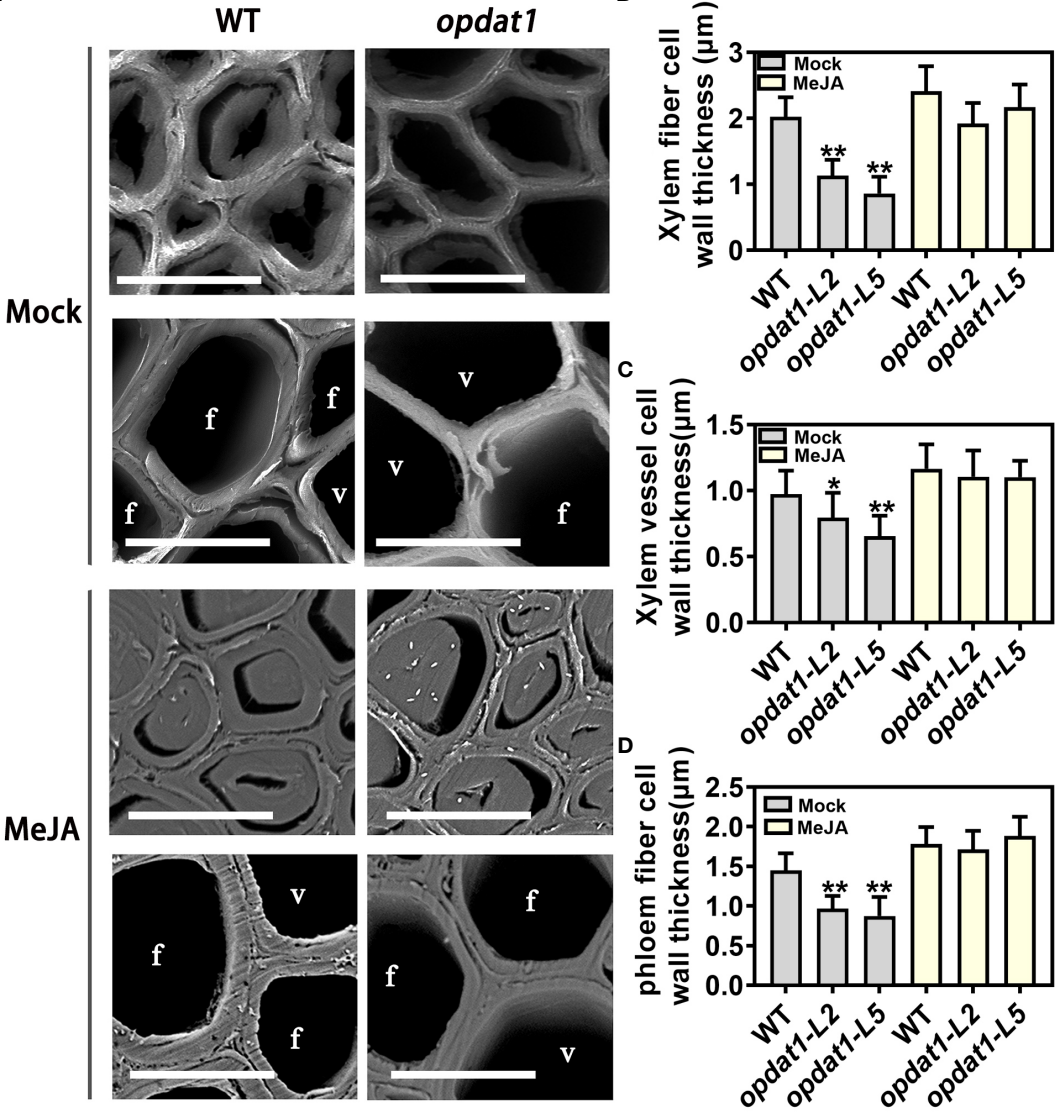
Section observation after MeJA treatment of *opdat1* mutant poplars. **(A)** Observation of poplar 5th internode stem sections by scanning electron microscope. Phloem cells above and xylem cells below in the treatment and control groups. **(B)** Xylem fiber cell secondary wall measurement. fiber cells. **(C)** Xylem vessel cell secondary wall measurement. v, vessel cell. **(D)** Phloem fiber cell secondary wall measurement. The scale bars = 10 μm. The error bars indicate the standard deviation of the data from the three biological replicates in the experiment. Asterisks indicate that the data from the experimental replicates show significant differences based on Student’s *t*-test (*P < 0.05; **P < 0.01).

To investigate the effects of JA on lignin deposition and SCW thickening in poplar, we treated wild-type (WT) poplar with MeJA solution for one month. Methyl jasmonate (MeJA) treatment significantly inhibited the growth of poplar stems (**Figure S2**). Toluidine blue and phloroglucinol-HCl staining of sections from the sixth internode stem revealed that MeJA treatment significantly increased the depth of xylem staining and SCW thickness (**Figure S3A**). Scanning electron microscopy also showed that MeJA treatment promoted the thickening of SCW in vessel and fiber cells (**Figures S3B-D**), which was consistent with the increased lignin content in treated poplar (**Figure S3E**). Furthermore, RT-qPCR analysis revealed that the expression levels of lignin synthase genes (*COMT2*, *CCoAOMT1*, *CCR2*, *CAld5H2*, *PAL1*, *CSE1*, *C4H2*, and *4CL5*), cellulose synthase gene (*CESA3A*), xylan synthase gene (*GT43B*), and some laccase genes (*LAC29*, *LAC30*, and *LAC41*) were significantly induced by MeJA treatment (**Figure S3F**). The application of MeJA to the *opdat1* mutant also significantly restored the phenotype of SCW thinning (**Figure 2**), suggesting that MeJA treatment promotes SCW thickening by inducing the expression of genes related to secondary wall biosynthesis.

### PtoJAZ5 is likely to be involved in regulating xylem development

It is well known that the degradation of JAZ proteins is the main mechanism of JA signaling in plants (Thines et al., 2007). To investigate the role of JA signaling in lignin deposition and SCW synthesis, we aimed to reduce JA signaling by overexpressing JAZ genes. We identified 12 JAZ proteins in poplar and constructed a phylogenetic tree of Arabidopsis and poplar JAZ protein sequences (**Figure S4A**). Poplar JAZ5 has a 58.48% amino acid sequence similarity to Arabidopsis AtJAZ1/2, and contains the conserved Tify and Jas domains with a conserved LPIARRA amino acid sequence in the Jas domain (**Figure S4B**). Previous studies have demonstrated that AtJAZ1 is involved in plant growth, development, and response to freezing and senescence (Robson et al., 2010; Zhou et al., 2017; Valenzuela-Riffo et al., 2020). Gene expression profile data of poplar JAZ proteins showed that *PtoJAZ5* is specifically expressed in the xylem (**Figures S4C, D**), indicating that PtoJAZ5 may play a role in the transcriptional regulation of JA signaling in the xylem region. Therefore, we cloned the poplar *PtoJAZ5* gene for further investigation.

### Subcellular localization of PtoJAZ5 and its expression pattern

**Figure 3:**
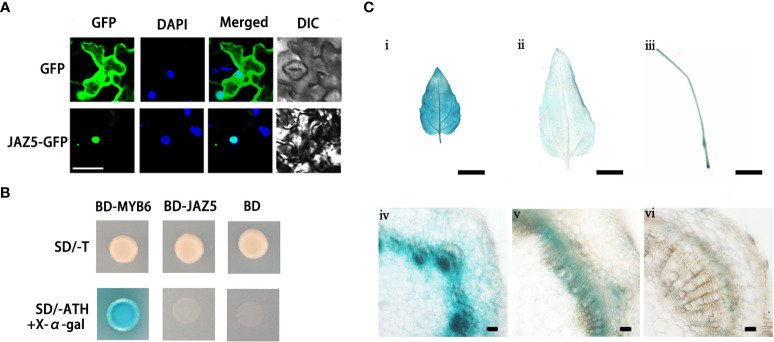
Analysis of subcellular localization, tissue expression and transcriptional activity of poplar JAZ5 protein. **(A)** Subcellular localization observation of JAZ5. DAPI is a nuclear dye; DIC indicates observation under white light. Scale bar: 50 μm. **(B)** Analysis of transcriptional activity of JAZ5. MYB6 is a transcriptional activator and was used as a positive control in this experiment (Wang et al., 2019) and BD is the empty vector for negative control. SD/-T represents one deficient yeast medium lacking tryptophan. SD/-ATH represents three deficient yeast medium lacking tryptophan, histidine and adenine. X-α-gal is a chromogenic substrate for yeast galactosidase (MEL1). **(C)** GUS staining for young leaves (і), old leaves (і), roots (ii), stem second internode (iii), stem fourth internode (iv), and stem base (v). scale bars: 0.5 cm (і-iii); 100 μm (iii-v).

To investigate the subcellular localization of PtoJAZ5, we generated a PtoJAZ5-GFP fusion protein driven by the *CaMV 35S* promoter and transiently expressed it in tobacco epidermal cells. The localization of the fusion protein was visualized by observing the GFP fluorescence signal. The results showed that PtoJAZ5 is primarily localized in the nucleus and co-localized with the DAPI signal, confirming that PtoJAZ5 is a nuclear-localized protein (**Figure 3A**). To further explore the transcriptional activity of PtoJAZ5, we fused PtoJAZ5 to the GAL4 DNA binding domain (BD) and transformed the recombinant plasmids (BD-PtoJAZ5) into yeast cells. The transformed yeast cells were able to grow on selective medium lacking tryptophan (SD/-T), but could not grow on medium lacking tryptophan, histidine and adenine (SD/-ATH), indicating that PtoJAZ5 does not have transcriptional activation activity (**Figure 3B**).

To investigate the spatiotemporal expression pattern of *PtoJAZ5*, we constructed a *PtoJAZ5* promoter-*GUS* fusion vector. GUS staining revealed that *PtoJAZ5* is mainly expressed in the cambium layer and developing xylem region, young leaves, roots, and stems of poplar (**Figure 3C**). These results suggest that PtoJAZ5 may play a role in the growth and development of xylem.

### Overexpression of *PtoJAZ5* inhibited SCW thickening in poplar and Arabidopsis

To determine the role of PtoJAZ5 in SCW formation, we overexpressed *PtoJAZ5* in poplar under the control of the *CaMV 35S* promoter. RT-qPCR assay was performed to select transgenic lines (Line 8 and Line 9) with high expression of *PtoJAZ5* for further experiments (**Figure S5D**). No significant difference in the appearance phenotype was observed between transgenic and WT poplars, except for a slight increase in height of some transgenic plants (**Figures S5A-C**). RT-qPCR results showed that the expression levels of JA signaling marker genes (*PR4*, *VSP1*, *VSP2*) were significantly down-regulated in *PtoJAZ5* overexpression plants (**Figure S5E**), indicating the suppression of JA signaling.

**Figure 4:**
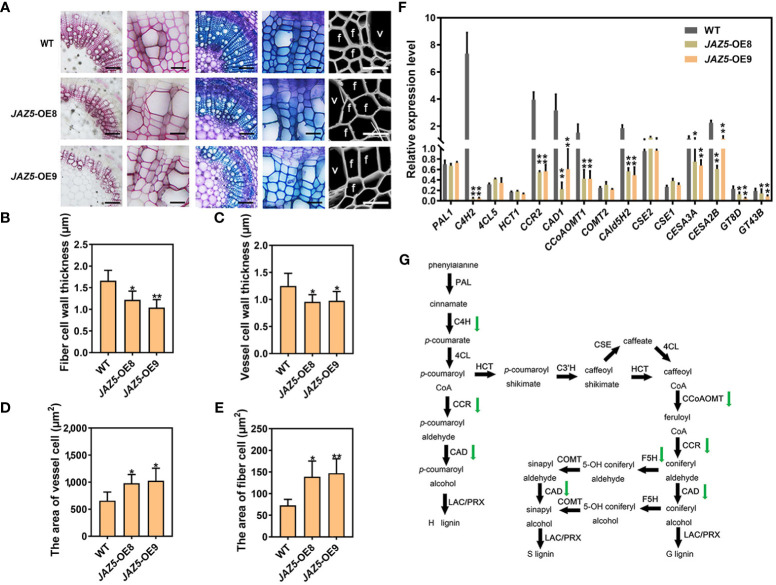
Reduced lignin deposition and secondary cell wall thinning during wood formation in *JAZ5*-OE transgenic poplar plants. **(A)** Section observation of wild-type and transgenic plants, from left to right, with Ph-cl, toluidine blue staining and scanning electron microscopy. **(B-E)** Statistical analysis of the stem region for fiber secondary wall, vessel cell secondary wall, vessel cell size and fiber cell size. Scale bars (Figure A) = 100 μm (columns 1, 3), 50 μm (columns 2, 4), 20 μm (columns 5). In the picture, f represents fiber cells and v represents vessel cells. OE8 and OE9 represent two different *JAZ5* overexpression lines. **(F)** RT-qPCR of secondary cell wall synthesis-related enzyme genes of stem in *JAZ5-OE* poplar, including lignin synthase genes (*PAL1, C4H2, 4CL5, HCT1, CCR2, CAD1, CCoAOMT1, COMT2, CAld5H2, CSE2* and *CSE1*), cellulose synthase genes (*CESA3A* and *CESA2B*) and xylan synthase genes (*GT8D* and *GT43B*). **(G)** Schematic diagram of the lignin synthesis pathway. The error bars indicate the standard deviation of the data from the three biological replicates in the experiment. Asterisks indicate that the data from the experimental replicates show significant differences based on Student’s *t*-test (*P < 0.05; **P < 0.01).

To further investigate the effect of PtoJAZ5 on SCW formation, we performed section staining and scanning electron microscopy on the sixth internode stem of transgenic poplar. In comparison to WT, a lighter staining color of the xylem region and a significant reduction in the thickness of vessel cells and fiber cells were observed in the *PtoJAZ5* overexpression plants compared with WT. However, the area of vessel cells and fiber cells was significantly increased (**Figures 4A-E**). RT-qPCR results showed that the expression levels of SCW biosynthase genes including lignin synthase genes (*C4H2*, *CCR2*, *CAD1*, *CCoAOMT1*, and *CAld5H2*), cellulose synthase genes (*CESA3A*, *CESA2B*), and xylan synthase genes (*GT8D* and *GT43B*) were significantly down-regulated in the transgenic plants (**Figures 4F, G**). Additionally, exogenous MeJA treatment significantly restored the phenotype of SCW thinning in transgenic poplars (**Figure S8**). These results indicate that the SCW thinning in *PtoJAZ5* overexpression poplar is mediated by a decrease in the expression level of SCW synthesis-related enzyme genes.

To examine the conservation of JAZ5 function, we also heterologously overexpressed *PtoJAZ5* in Arabidopsis. Analysis of stem sections of transgenic Arabidopsis showed that the autofluorescence intensity of lignin was significantly reduced, and the color of toluidine blue staining and phloroglucinol-HCl staining in stem of Arabidopsis overexpressing *PtoJAZ5* was lighter compared to WT. Scanning electron microscopy revealed significant thinning of SCW in transgenic Arabidopsis (**Figure S6**). Consistent with the phenotypic observation, RT-qPCR results revealed that transcript levels of SCW synthase genes in transgenic Arabidopsis were significantly reduced (**Figure S7**). These results suggest that PtoJAZ5 is able to inhibit lignin deposition and SCW thickening in Arabidopsis, indicating its conserved function.

### PtoJAZ5 is able to interact with SCW biosynthetic-related NAC/MYB transcription factors

**Figure 5:**
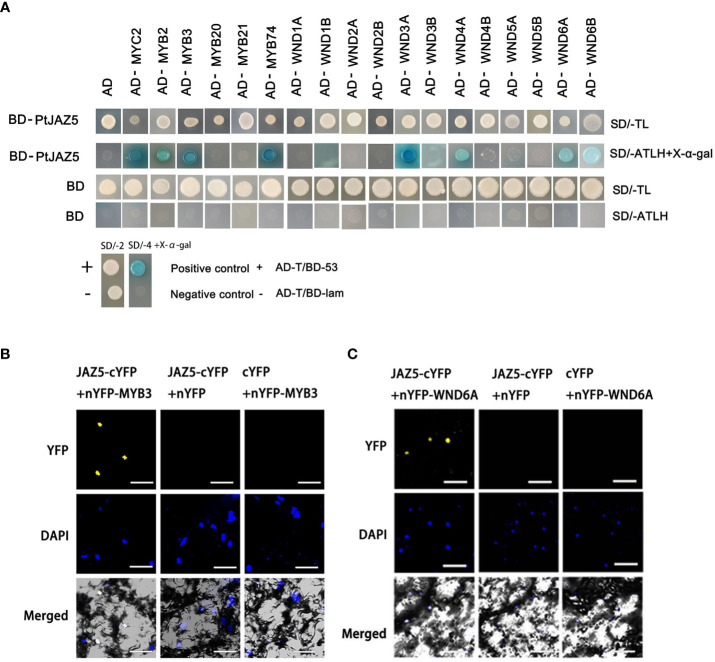
Interaction analysis of JAZ5 with NAC and MYB transcription factors from poplar via Y2H assay. **(A)** AD and BD denote PGADT7 and PGBKT7 vectors, respectively. The BD vector fusion protein JAZ5 was constructed, and the AD vector fusion protein included JA signaling transcription factor MYC2 and key regulators of secondary wall synthesis (MYB transcription factor MYB2/3/20/21/74 and NAC transcription factor WND1A/1B/2A/2B/3A/3B/4A/4B/5A/5B/6A/6B). AD-T and BD-53 vectors are positive controls; AD-T and BD-lam, BD and AD empty vectors are negative controls. SD/-TL represents a two-deficient yeast medium lacking tryptophan and leucine. SD/-ATLH represents a four-deficient yeast medium lacking tryptophan, leucine, histidine and adenine. X-α-gal is a chromogenic substrate for yeast galactosidase (MEL1). **(B, C)** Bimolecular Fluorescent Complimentary (BiFC), the C-terminal fusion protein JAZ5-cYFP expression vector of JAZ5-linked yellow fluorescent protein (YFP) and the N-terminal fusion proteins MYB3-nYFP and WND6A-nYFP expression vectors of MYB3- and WND6A-linked YFP were constructed, and the C-terminal and N-terminal empty expression vectors of YFP were used as negative controls to transform tobacco leaves for transient expression. The scale bars = 10 μm.

Intensive studies have shown that JA signaling is mediated by the interaction of JAZ proteins with transcription factors, which results in the suppression or activation of downstream genes depending on JA levels (Chini et al., 2007; Thines et al., 2007; Katsir et al., 2008; Pauwels et al., 2010). Using yeast two-hybrid (Y2H) experiments, we searched for SCW synthesis-related transcription factors that interact with JAZ5. The Y2H results revealed that PtoJAZ5 interacted with several SCW synthesis switch masters, such as MYB2, MYB3, MYB74, WND3A, WND4A, WND6A, and WND6B (**Figure 5A**). Previous studies have shown that poplar MYB3/2/20/21, which are mainly expressed in the developing xylem region, act as secondary switches in the regulatory network of SCW synthesis (Wilkins et al., 2009; Mccarthy et al., 2010). The WNDs, belonging to the NAC gene family, are first-order switches in the regulatory network of SCW synthesis in poplar (Zhong et al., 2010; Zhong et al., 2011). Based on the gene expression analysis and Y2H experiment, we selected MYB3 and WND6A as representative transcription factors to further investigate the function of PtoJAZ5 (**Figure 5A** and **Figure S9**). We constructed PtoJAZ5-PXY104, a C-terminal fusion expression vector of PtoJAZ5 with yellow fluorescent protein (PtoJAZ5-cYFP), and PXY106-MYB3 and PXY106-WND6A, fusion expression vectors of the N-terminal of YFP with MYB3 and WND6A (nYFP-MYB3 and nYFP-WND6A), for fluorescent bimolecular complementation experiments (BiFC). The combination of PtoJAZ5/MYB3 and PtoJAZ5/WND6A showed a yellow fluorescent signal in tobacco leaves, while the other two negative controls showed no signal when observed under laser confocal electron microscopy (**Figures 5B, C**). These results suggest that PtoJAZ5 interacts with MYB3 and WND6A in plant cells.

### JAS domain is required for the interaction of PtoJAZ5 with MYB3/WND6A

To identify the core amino acid sequence fragments of PtoJAZ5 that interact with MYB3 or WND6A, we constructed a yeast fusion expression vector of PGBKT7 by truncating PtoJAZ5 into different structural domains, including JAS domain, Tify domain, and N-terminal domain, based on the amino acid sequence specificity of PtoJAZ5. The Y2H assays showed that proteins containing the JAS domain could interact with MYB3 or WND6A, and yeast transformants grew normally on SD/-ATLH-deficient medium. Conversely, yeast transformants failed to grow when the JAS domain was absent (**Figures S10A, B**). Therefore, we conclude that the JAS domain is the necessary core sequence for PtoJAZ5 to interact with MYB3 or WND6A.

Previous studies have revealed that the JAS structural domain mediates the degradation of JAZ protein by JA receptor COI1 (Sheard et al., 2010). Based on amino acid sequence analysis of PtoJAZ5, we mutated the core sequence of the JAZ degradation domain, LPIAR, to LPIGA, to construct a PtoJAZ5 mutant protein (PtoJAZ5m). Using a Y2H assay to verify whether PtoJAZ5m protein could still interact with MYB3/WND6A, and found that this sequence mutation did not affect the interaction of PtoJAZ5 with MYB3 or WND6A (**Figure S10C**). Next, we investigated whether the LPIAR sequence mutation affected the degradation of PtoJAZ5. We constructed PtoJAZ5-GFP and PtoJAZ5m-GFP fusion protein expression vectors and transformed them into Arabidopsis using floral dip method. Transgenic Arabidopsis seedlings were treated with MeJA solution and observed under a fluorescence microscope. The results showed that the green fluorescence signal was diminished or even disappeared in transgenic Arabidopsis roots expressing PtoJAZ5 but not in those expressing PtoJAZ5m (**Figures S11A, B**). These findings indicate that LPIAR is the core sequence of the degradation domain of PtoJAZ5, which responds to JA signals.

### The interaction between PtoJAZ5 and MYB3/WND6A inhibited the expression of SCW synthesis-related genes

**Figure 6:**
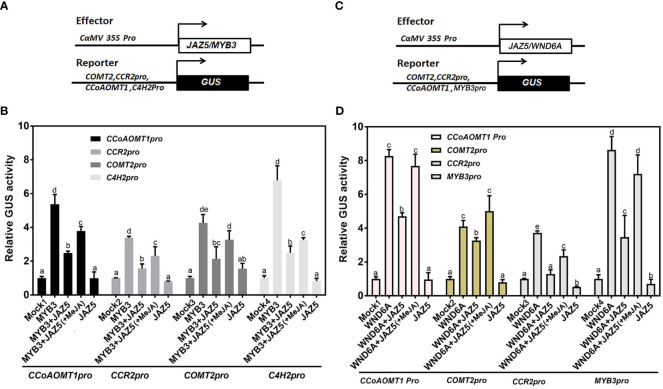
JAZ5 represses transcriptional activation of downstream genes by MYB3 and WND6A. **(A, B)** JAZ5 represses the transcriptional activation of lignin synthesis-related enzyme genes by MYB3. **(C, D)** JAZ5 represses the transcriptional activation of lignin synthesis-related enzyme genes and *MYB3* via WND6A. Experiments were performed using tobacco leaf transient expression technology, in which the promoter-linked *GUS* reporter gene expression vectors of *CCoAOMT1, CCR2, COMT2, C4H2*, and *MYB3* were co-transformed with the expression vectors of effector proteins JAZ5 and MYB3/WND6A to detect GUS protein activity. MeJA in parentheses indicates exogenous MeJA treatment. The error bars indicate the standard deviation of the data from the three biological replicates. Different lowercase letters represent significant differences, and one-way ANOVA was used for statistical analysis of differences.

To investigate whether the interaction between PtoJAZ5 and MYB3/WND6A affects downstream SCW synthase gene expression levels, we used the promoters of *CCoAOMT1*, *CCR2*, *COMT2*, and *C4H2* to control the expression of *GUS* reporter genes. The generated reporter vectors were co-transformed into tobacco leaf epidermal cells with overexpression vectors, enabling transactivation analysis. Our findings demonstrate that MYB3 or WND6A alone significantly activated the expression of *GUS* genes under the control of the aforementioned promoters. However, this activation was inhibited by PtoJAZ5. Upon MeJA treatment, the repressive effect of PtoJAZ5 on MYB3 and WND6A was alleviated (**Figure 6**). Thus, our results suggest that PtoJAZ5 regulates SCW biosynthesis via interacting with MYB3 or WND6A, and that this regulatory mechanism is modulated by JA signaling.

## Discussion

In woody plants, lignin deposition and SCW thickening are crucial for providing mechanical support and physical defensive structures. Various plant hormones, such as auxin, gibberellin, ethylene, abscisic acid, and JA, play important roles in this process (Burger and Chory, 2019; Buttò et al., 2019; Jewaria et al., 2021). Despite their importance, the mechanism by which JA signaling regulates plant SCW development remains unclear.

Previous studies have shown that exogenous application of MeJA treatment promotes thickening of xylem SCWs and ectopic deposition of lignin in Arabidopsis stems (Sehr et al., 2010). However, its molecular mechanism remains unknown and has not been studied in woody plants. In this study, we provide evidence that MeJA treatment induces elevated expression levels of genes related to SCW synthesis, thereby promoting lignin deposition and SCW thickening in poplar stems (**Figure S3**). Additionally, we confirmed that lower JA levels in poplar led to thinning of the SCW using section observation of the *opdat1* mutant and exogenous JA reversion experiments (**Figures 1**, **2**). These results indicate that proper JA content is necessary for SCW development in poplar.

Numerous studies have confirmed that JA mainly functions through the regulation of the repressor JAZ protein and the core transcription factor MYC2 (Chini et al., 2007; Pauwels and Goossens, 2011; Breeze, 2019). For example, MYC2, MYC3, and MYC4 can bind to JAZs to regulate root growth, insect defense, leaf senescence and wounding response in plants (Sasaki-Sekimoto et al., 2013; Zhuo et al., 2020). JAZ1/8/11 regulates anthocyanin synthesis by inhibiting the action of MBW complex (Qi et al., 2011). Therefore, in order to further investigate the mechanism of JA in secondary cell wall development, we overexpressed *PtoJAZs* to inhibit the JA signaling pathway. We identified 12 poplar JAZ proteins by sequence alignment with Arabidopsis JAZ proteins, and selected PtoJAZ5, which was specifically and highly expressed in stems, as a representative for further study. A plant expression vector carrying *PtoJAZ5*-OE cassettes was constructed and transgenic poplar plants were generated (**Figures S5**, **S6**). Histochemical staining of toluidine blue and phloroglucinol-HCl, and scanning electron microscope observation showed that constitutive expression of *PtoJAZ5* inhibited SCW thickening and lignin deposition in poplar (**Figure 4**) and Arabidopsis (**Figures S6**, **S7**). RT-qPCR analysis revealed that overexpression of *PtoJAZ5* resulted in decreased expression levels of SCW biosynthetic genes for lignin, cellulose and xylan (**Figures 4F, G**). Similar results were obtained from *PtoJAZ5*-OE Arabidopsis (**Figure S7**). These results are consistent with the findings in Arabidopsis (Sehr et al., 2010) and demonstrate the regulatory role of JA in secondary cell wall development in poplar.

In conclusion, our study provides evidence that JA promotes lignin deposition and SCW thickening in poplar trees. Specifically, we found that JAZ proteins, such as PtoJAZ5, act as inhibitors of JA signal transduction and can suppress the expression and activation of SCW synthase genes by interacting with the SCW synthesis switch factor WND/MYB, such as WND6A/MYB3 (**Figures 5**, **6**). This leads to a reduction in SCW synthesis and lignin deposition in poplar trees. JAZ proteins may not only interact with MYC2 to regulate growth and development and secondary wall synthesis (Zhang et al., 2018; Luo et al., 2022), but they also directly interact with secondary wall synthesis switch factors to influence secondary wall deposition. These findings extent our understanding of the regulatory network of JA in coordinating plant growth and development, as well as defense responses.

## Data availability statement

The original contributions presented in the study are included in the article/**Supplementary Material**. Further inquiries can be directed to the corresponding authors.

## Author contributions

XZ, CX, and KL designed the work. XZ, XJ, ZL and QS performed experiments and analyzed data. XZ, and KL drafted the manuscript. CX and KL revised the manuscript. All authors contributed to the article and approved the submitted version.
