# Supplementary material for: Jasmonic acid regulates lignin deposition in poplar through JAZ5-MYB/NAC interaction
Source: Front Plant Sci. 2023 Jul 21;14:1232880. doi: 10.3389/fpls.2023.1232880 (PMC10401599; doi:10.3389/fpls.2023.1232880)
Supplement: Supplementary file 1 [file DataSheet_1.docx]

**Supplemental Information**

**Jasmonic acid regulates lignin deposition in poplar through JAZ5-MYB/NAC interaction**

Xin Zhao^1,2^, Xuemei Jiang^1,3^, Zeyu Li^1,3^, Qin Song^1,3^, Changzhen Xu^1,3, *^, Keming Luo^1,3, *^

^1^ Chongqing Key Laboratory of Plant Resource Conservation and Germplasm Innovation, School of Life Sciences, Southwest University, Chongqing 400715, China

^2^Lab of Plant Cell Engineering, Southwest University of Science and Technology, Mianyang, Sichuan, China

^3^ Key Laboratory of Eco-environments of Three Gorges Reservoir Region, Ministry of Education, School of Life Sciences, Southwest University, Chongqing 400715, China

***Corresponding author：Keming Luo.**

**Email:** [**luokeming@hotmail.com**](mailto:luokeming@hotmail.com)

***Corresponding author：Changzhen Xu.**

**Email: xucz@swu.edu.cn**

**Supplementary Figures: 10**

**Supplementary Tables: 1**


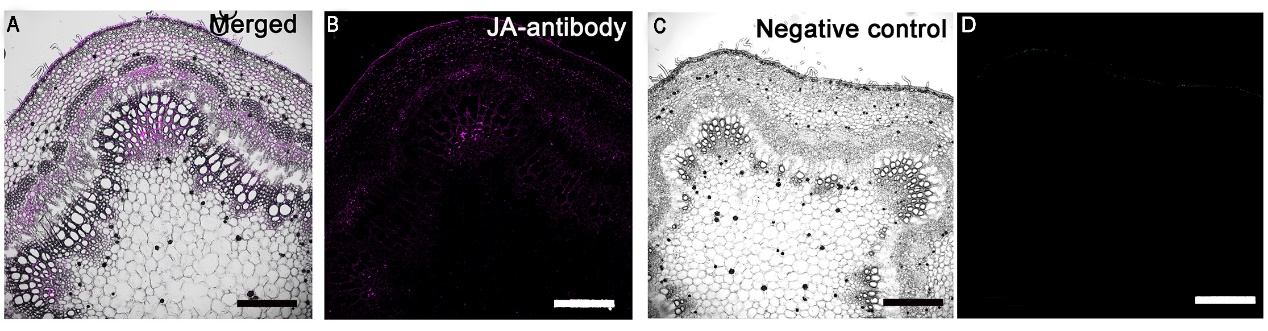


**Figure S1. Immunocytochemical detection of JA of wild type poplar.**

(A and B) Immunofluorescence of JA of the 5th internode stem section in poplar, A is the overlapping picture under fluorescent and bright field, B is the fluorescent picture. (C and D) JA immunofluorescence negative control, stem samples without added primary antibody were used as negative control, C is the overlapping picture under fluorescent and bright field, D is the fluorescent picture. Scale bar = 100 μm.


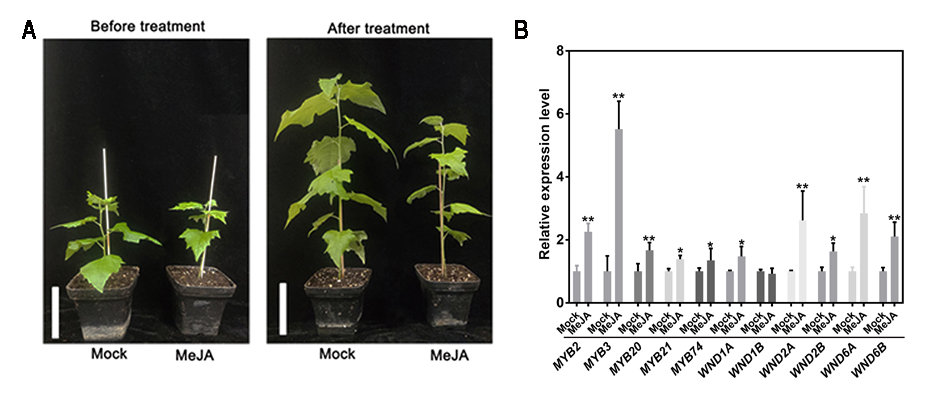


**Figure S2. MeJA treatment of wild-type poplar.**

(A) Overall phenotypic observation of poplar trees after MeJA treatment for 30 d. Scale bars: 9cm. (B) Gene expression levels of secondary wall synthesis-related transcription factors (includng *MYB2/3/20/21/74* and *WND1A/1B/2A/2B/6A/6B*) in stems were examined 1 day after MeJA treatment in poplar. The error bars indicate the standard deviation of the data from the three biological replicates in the experiment. Asterisks indicate that the data from the experimental replicates show significant differences based one-way ANOVA analysis (* *P* < 0.05; ** *P* < 0.01).


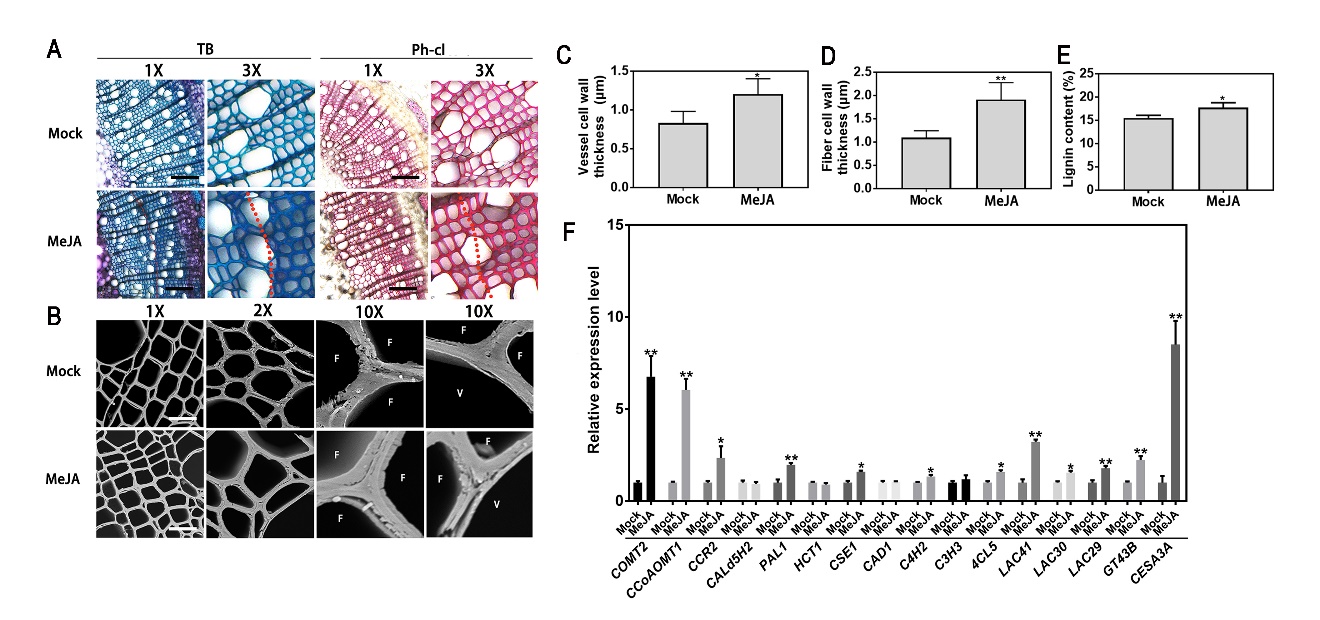
**Figure S3. Exogenous application of MeJA promotes lignin deposition and secondary cell wall thickening in poplar.**

(A) Staining of poplar stem sections, blue represents toluidine blue staining, red represents Phloroglucinol-HCl staining, and the rightmost image is a threefold magnification of the MeJA-treated group sections. The red dashed line indicates the dividing line of xylem growth before and after MeJA treatment. The scale bar is 100 μm. (B) Scanning electron microscope images of poplar stem sections. 1X, 2X, 3X and 10X stand for 1X, 2X, 3X and 10X magnification. In the picture, F indicates fiber cells, V indicates vessel cells. The scale bar represents 20 μm. (C and D) Measurement of secondary cell wall thickness of stem vessel cells and fiber cells. (E) Lignin content determination. (F) Expression levels of secondary wall synthase genes (including lignin synthase genes *COMT2, CCoAOMT1, CCR2, CAld5H2, PAL1, CSE1, C4H2,* and *4CL5*, cellulose synthase gene *CESA3A*, xylan synthase gene *GT43B*, and laccase genes *LAC29, LAC30*, and *LAC41*) in stems were examined after MeJA treatment for 1 day in poplar. The error bars indicate the standard deviation of the data from the three biological replicates in the experiment. Asterisks indicate that the data from the experimental replicates show significant differences based one-way ANOVA analysis (** P* < 0.05; *** P* < 0.01).


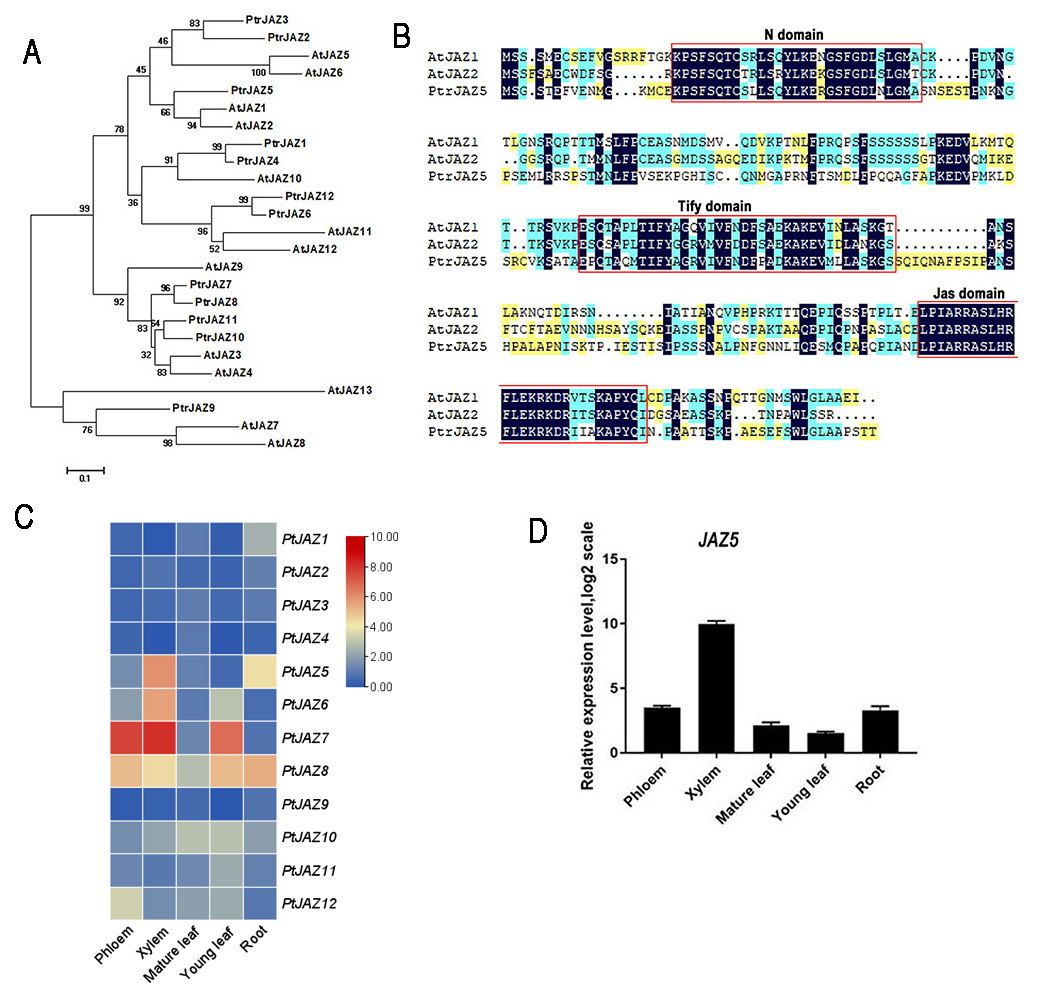


**Figure S4. Phylogenetic tree analysis and gene expression pattern of *JAZ* in poplar.**

(A) Phylogenetic tree analysis of JAZ protein amino acid sequences derived from P. trichocarpa (Ptr), A.thaliana (At). (B) Amino acid sequence comparison of poplar JAZ5 with Arabidopsis AtJAZ1/2. Red boxes represent the amino acid sequences corresponding to the structural domains of the proteins. (C) Analysis of gene expression patterns of all 12 JAZ proteins in poplar root, young leave, mature leaf, stem xylem and phloem. The Expression data were obtained from previous study (Wang et al. 2017). The vertical color scale to the right of the figure displays log2 expression values, red denotes a high level of transcript abundance, blue denotes a low level. (D) RT-qPCR analysis of *JAZ5* in poplar root, young leave, mature leaf, xylem and phloem.

Wang Y, Pan F, Chen D, Chu W, Liu H and Xiang Y (2017) Genome-wide identification and analysis of the Populus trichocarpa TIFY gene family. Plant Physiol Biochem 115:360-371.


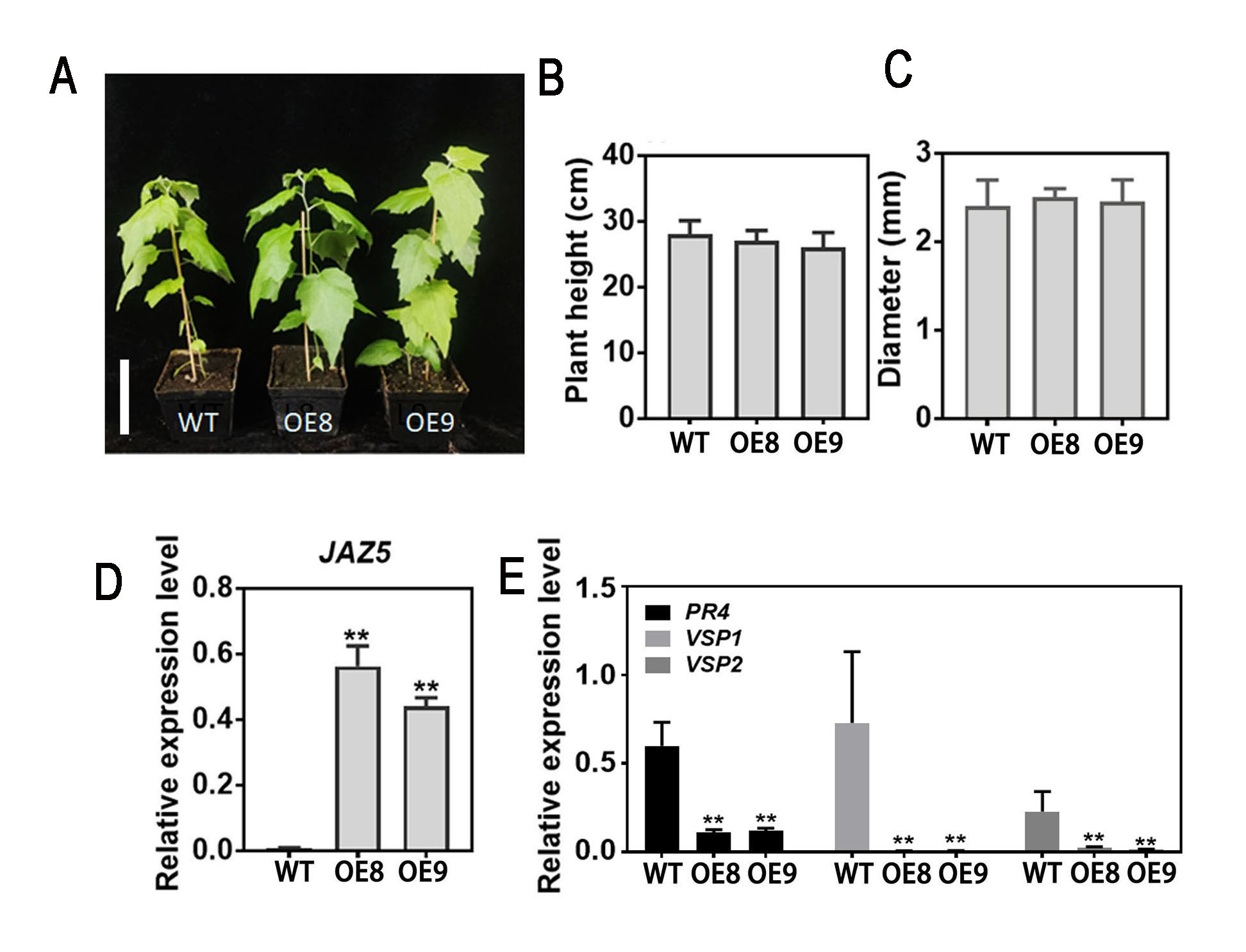


**Figure S5. Phenotype observation of *JAZ5-OE* transgenic poplar.**

(A) Morphological phenotypes observation of *JAZ5-OE* poplar plants. Scale bars = 10 cm. (B) Plant height statistics. (C) Plant diameter statistics. (D) Detection of *JAZ5* expression levels. (E) Expression levels of JA signal transduction marker genes (*VSP1, VSP2* and *PR4*). OE8 and OE9 represent two different *JAZ5* overexpression lines. The error bars indicate the standard deviation of the data from the three biological replicates in the experiment. Asterisks indicate that the data from the experimental replicates show significant differences based on Student's *t*-test (** P* < 0.05; *** P*< 0.01).


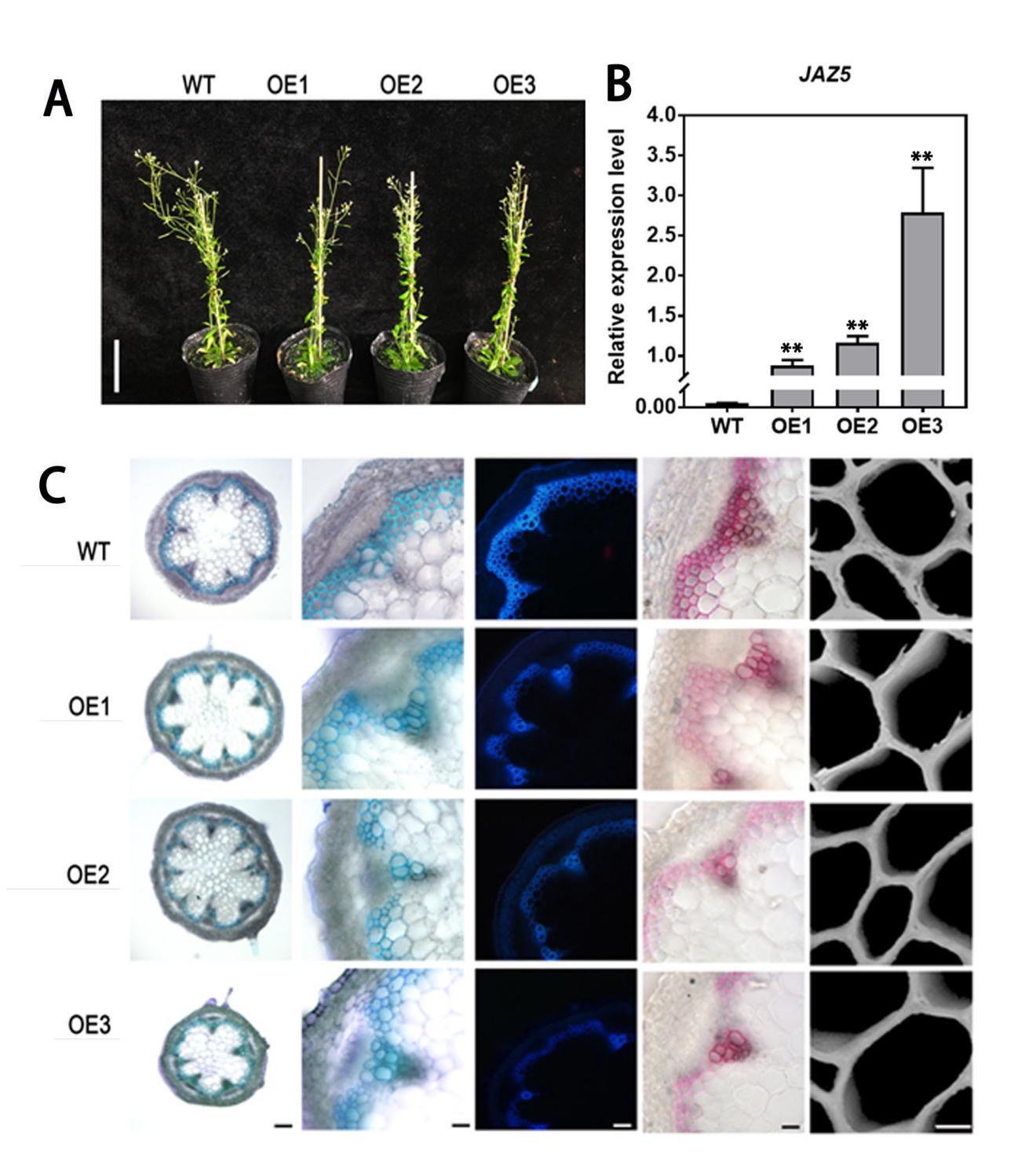


**Figure S6. Overexpression of poplar *JAZ5* in Arabidopsis inhibits secondary wall synthesis.**

(A) Morphological phenotypes of Arabidopsis at six weeks of growth. Scale bar = 10 cm. (B) Poplar *JAZ5* expression level assay. (C) Cross sections of the basal stems from *JAZ5-OE* plant. From left to right, xylem toluidine blue staining, lignin autofluorescence, phloroglucinol-HCl staining, and fiber cell observation by scanning electron microscope. Scale bars = 200 μm (column 1); 50 μm (columns 2, 3, and 4); 10 μm (column 5). OE1, OE2 and OE3 represent three different *JAZ5* overexpression lines. The error bars indicate the standard deviation of the data from the three biological replicates in the experiment. Asterisks indicate that the data from the experimental replicates show significant differences based on Student's *t*-test (** P* < 0.05; *** P* < 0.01).


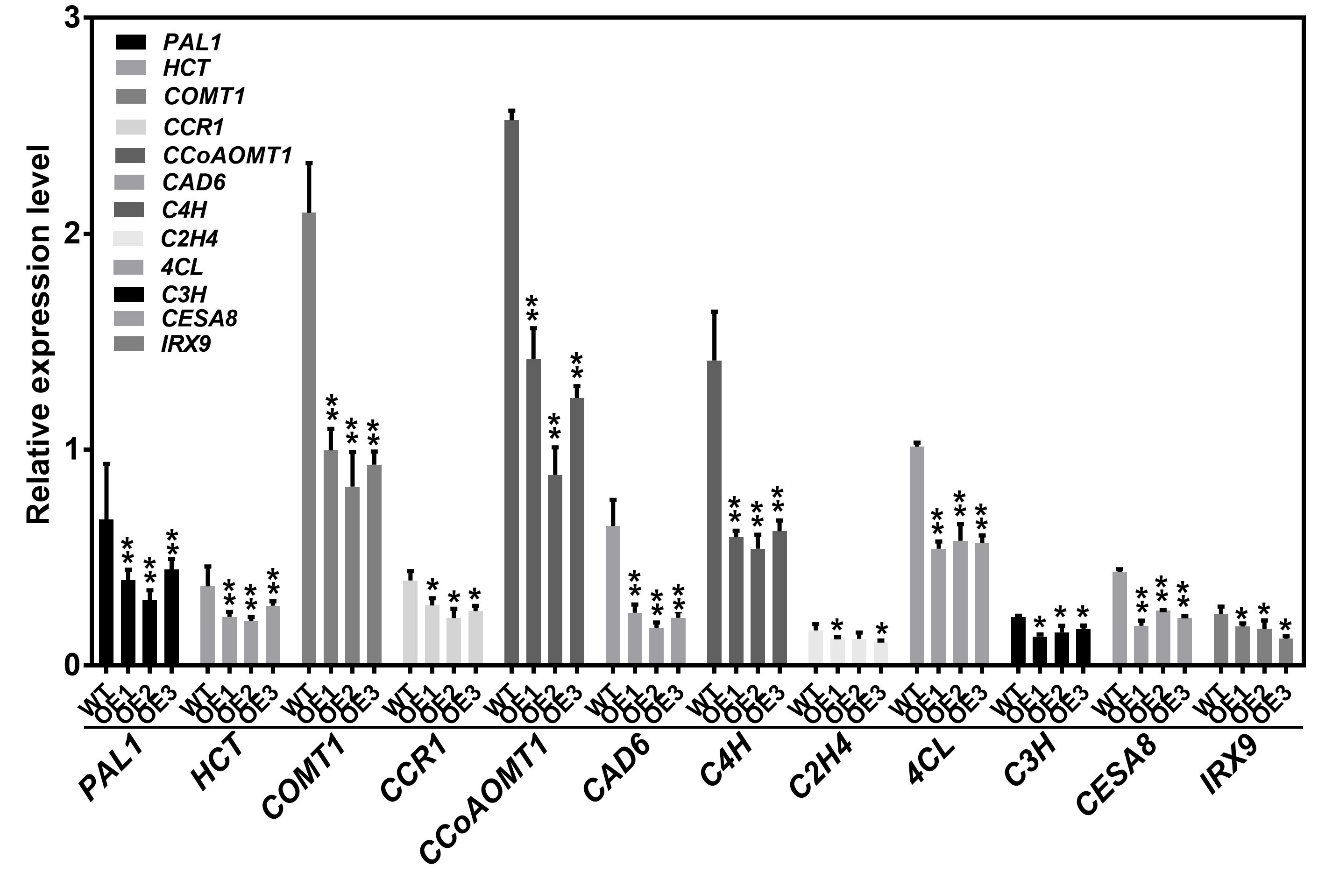


**Figure S7. Analysis of the expression of secondary wall biosynthetic related genes in *PtoJAZ5-OE* Arabidopsis.**

RT-qPCR was performed to detect the expression levels of key enzyme genes for secondary wall synthesis, including lignin synthase genes (*PAL1, HCT, COMT1, CCoAOMT1, CAD6, C4H, 4CL* and *C3H*), cellulose synthase gene *CESA8* and xylan synthase gene *IRX9*. OE1, OE2 and OE3 represent three different *PtoJAZ5* overexpression lines. The error bars indicate the standard deviation of the data from the three biological replicates in the experiment. Asterisks indicate that the data from the experimental replicates show significant differences based on Student's *t*-test (** P* < 0.05; *** P* < 0.01).


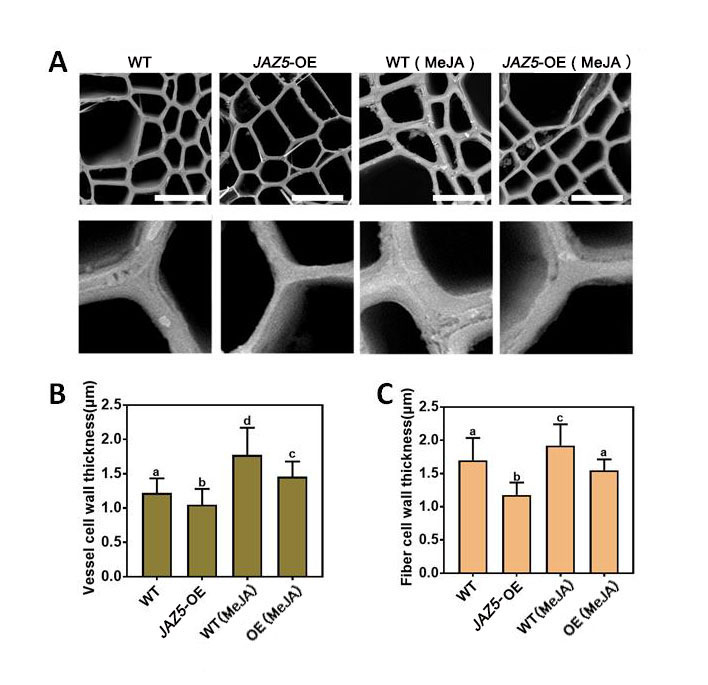


**Figure S8. Effect of exogenous MeJA treatment on the secondary cell wall of *JAZ5*-OE poplar.**

(A) Scanning electron microscopic observation of stem cross sections. Scale bar = 50 μm, the second row is magnified five times. (B) Vessel cell secondary wall thickness measurement. (C) Fiber cell secondary wall thickness measurement. The error bars indicate the standard deviation of the data from the three biological replicates in the experiment. Lowercase letters represent significant difference analysis. One-way ANOVA analysis was performed to evaluate statistical significance.


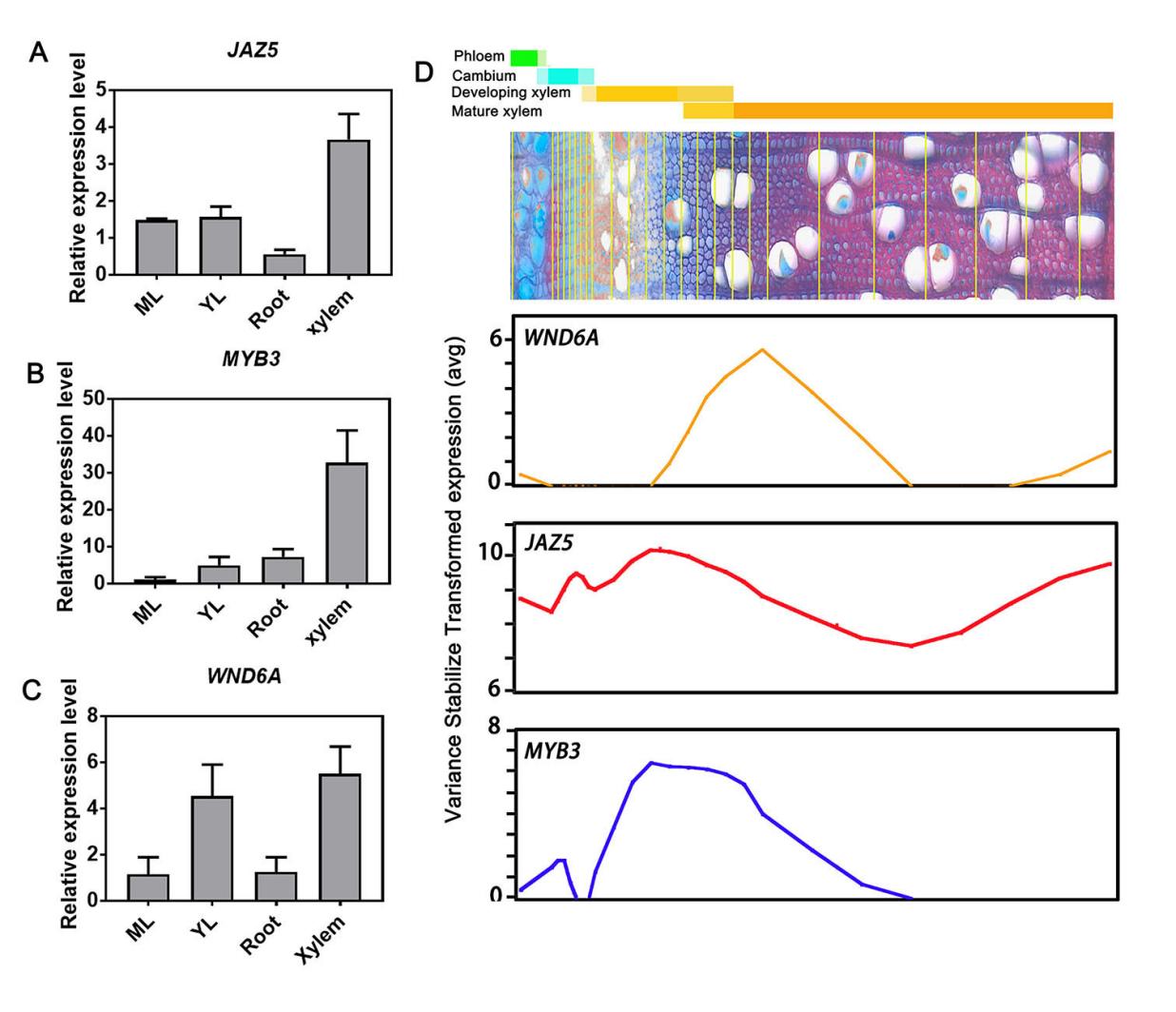


**Figure S9. Expression pattern of *MYB3, WND6A* and *JAZ5* in poplar.**

(A, B and C) RT-qPCR was performed to detect the expression levels of *MYB3, WND6A* and *JAZ5* in mature leaves, young leaves, roots and xylem of poplar. (D) Expression pattern of *MYB3, WND6A* and *JAZ5* in phloem, cambium, developing xylem and mature xylem in stem obtained from Aspwood website database query (http://aspwood.popgenie.org/aspwood-v3.0/).


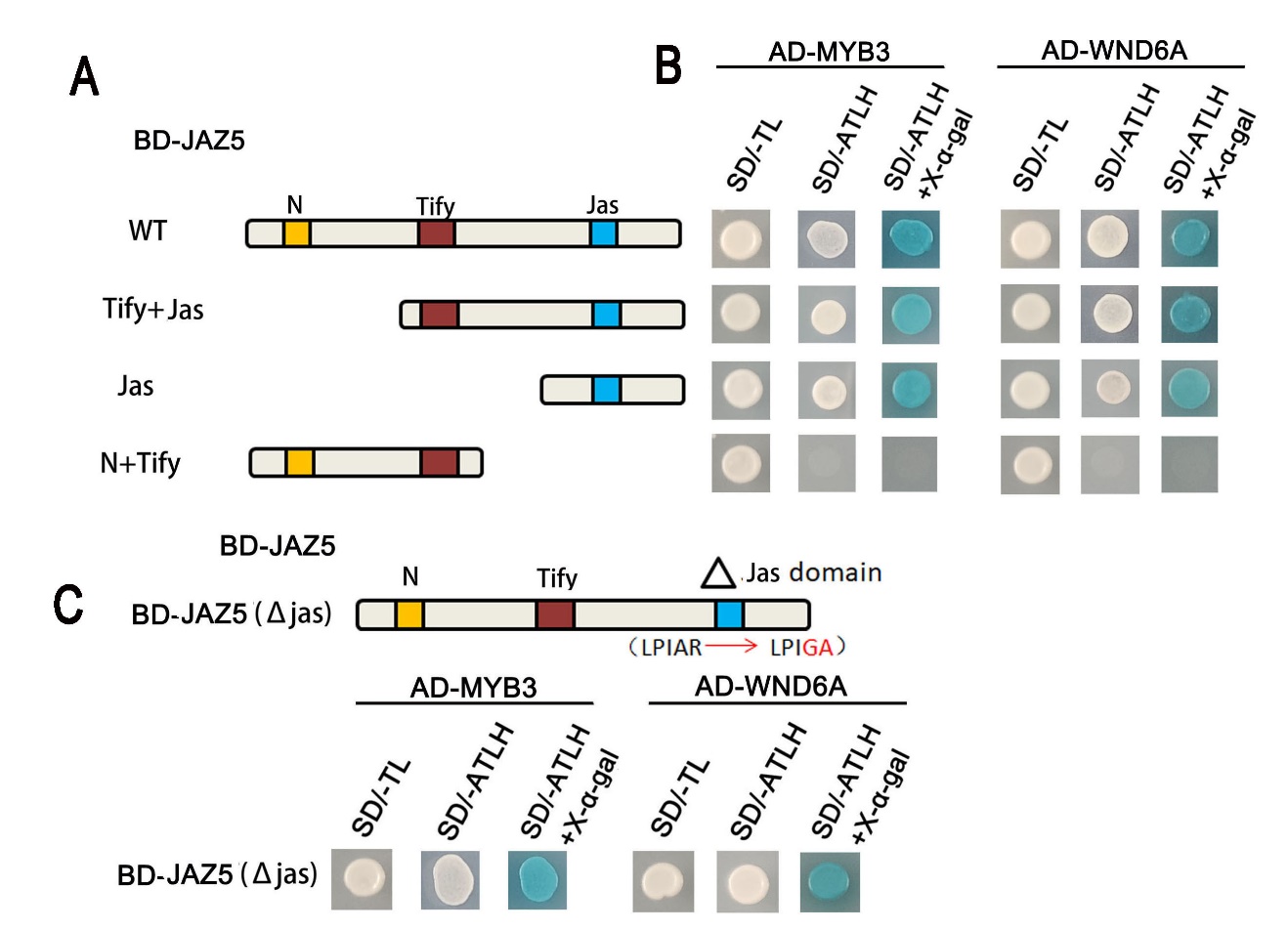


**Figure S10. Truncation and mutation analysis of JAZ5 amino acid sequence.**

(A) The schematic diagram of JAZ5 amino acid sequence truncation, WT (full length, 1-276), Tify+Jas (112-276), Jas (177-276), and N+Tify (1-176), respectively. (B) The results of yeast two-hybrid of JAZ5 truncated protein with WND6A and MYB3, respectively. (C) Analysis of amino acid sequence mutations in the JAZ5 degron, the JAZ5 amino acid sequence LPIAR was mutated to LPIGA (JAZ5m), and then JAZ5m was subjected to Y2H with MYB3/WND6A, below are the results of yeast two-hybrid of JAZ5m protein with WND6A and MYB3, respectively. SD/-TL represents a two-deficient yeast medium lacking tryptophan and leucine. SD/-ATLH represents a four-deficient yeast medium lacking tryptophan, leucine, histidine and adenine. X-α-gal is a chromogenic substrate for yeast galactosidase (MEL1).


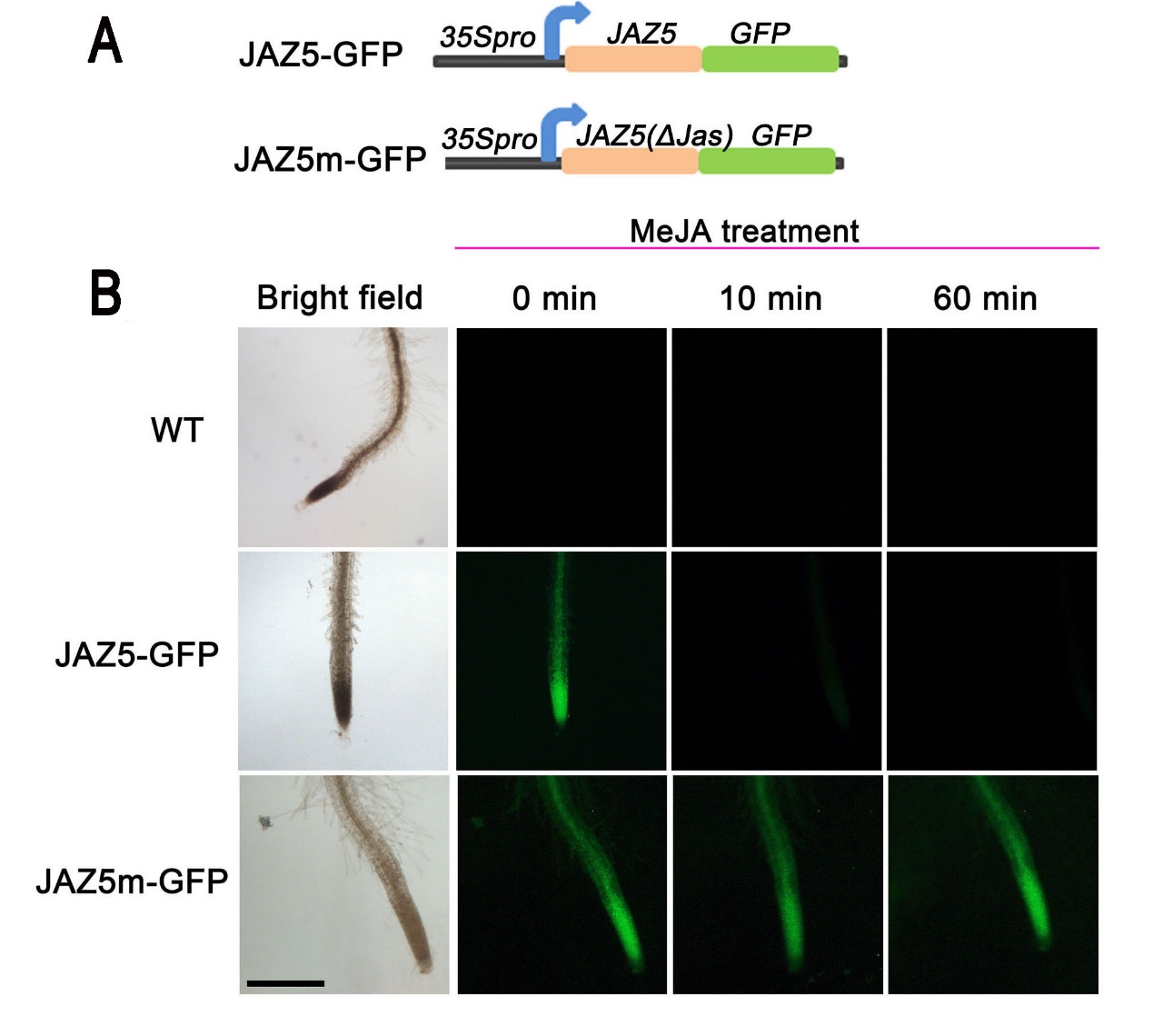


**Figure S11. Amino acid core sequence analysis of JAZ5 degron.**

(A) JAZ5/JAZ5m and GFP fusion protein expression vector. (B) The fluorescence signal of roots of transgenic Arabidopsis expressing GFP fusion proteins of JAZ5 and JAZ5m was observed to detect changes in fluorescence intensity after MeJA treatment. The scale bars = 1mm.

**Table S1. All primers used in this study.**

| **Primer name** | **Gene ID** | | | **Sequences** |
| --- | --- | --- | --- | --- |
| **RT-qPCR** | | | | |
| Q-JAZ1-F | Potri.001G062500 | | | CCTAATATGATGATCCCTCCCG |
| Q-JAZ1-R |  |  |  | CAAAATGTTCTCCGCCTTGTCT |
| Q-JAZ2-F | Potri.001G166200 | | | TTACAATCTGAAGCGCCTAACG |
| Q-JAZ2-R |  |  |  | TTTCCTCTTGGAGAAGAACCGA |
| Q-JAZ3-F | Potri.003G068900 | | | CTTCAAGGCAAAACGCTATGG |
| Q-JAZ3-R |  |  |  | CATTTGGGCGGTTTCAGGAT |
| Q-JAZ4-F | Potri.003G165000 | | | CTGCCTCCATTTCCTGCTTTG |
| Q-JAZ4-R |  |  |  | AAAATGTTCTCCGCCTTGTCT |
| Q-JAZ5-F | Potri.006G139400 | | | GGCTGACAAGGCTAAGGAAGTG |
| Q-JAZ5-R |  |  |  | TTGGCTGGAATAGAAGGAAAGG |
| Q-JAZ6-F | Potri.006G217200 | | | TGGTGGAAGTGTTGTCGTGTTT |
| Q-JAZ6-R |  |  |  | ATAGGCTTGGGCATGTGGG |
| Q-JAZ7-F | Potri.008G133400 | | | AACCACAGGAGCTTTGACCACT |
| Q-JAZ7-R |  |  |  | CGAGCCTGAGGGACAGATGG |
| Q-JAZ8-F | Potri.010G108200 | | | TGAATCTGATGCCTTGGTGTCC |
| Q-JAZ8-R |  |  |  | TGAAGAAAGGGTTGCCCGTAG |
| Q-JAZ9-F | Potri.011G083900 | | | AGTCGGGTCGGAGCCATCT |
| Q-JAZ9-R |  |  |  | TCGCTTCTTGAGGAACCTTTGT |
| Q-JAZ10-F | Potri.012G044900 | | | CGGTGGGTCTTCTGGAACG |
| Q-JAZ10-R |  |  |  | CGCTGACAGGTGGTCTACGAAT |
| Q-JAZ11-F | Potri.015G035800 | | | ACCCCGAGTTCCGTCATTG |
| Q-JAZ11-R |  |  |  | GTCCCAGAAGAGCCACCATTT |
| Q-JAZ12-F | Potri.018G047100 | | | AAGAAGCGACGGGACAGGTT |
| Q-JAZ12-R |  |  |  | TGGTGGAGGTGCGGCACTAAT |
| Q- MYC2-F | Potri.003G165000 | | | CTCTTGGGCTAAAGGCGAGT |
| Q- MYC2-R |  |  |  | GTCATACGTACGTTGCCATCC |
| Q- JMT-F | Potri.005G230100 | | | ACAAGGTTTGCTTGTGTCCA |
| Q- JMT-R |  |  |  | TCCTAGATCCAGTAAATCTTCATGT |
| Q- JAR1-F | Potri.014G095500 | | | CAGCAAGGTGCAGCAAATCC |
| Q- JAR1-R |  |  |  | AAACGGGCTAAGGAAAGTACC |
| Q- VSP2-F | Potri.002G257700 | | | TGGGCAGGTCTAGTTGTTGTC |
| Q- VSP2-R |  |  |  | CGGACGAAATGCCAACCAAG |
| Q- PR4-F | Potri.013G041600 | | | TGGCCAAAGCTGCGATGAAT |
| Q- PR4-R |  |  |  | AGTCCCAATTAGGCTCGGTT |
| Q-WND6A-F | Potri.013G113100 | | | ACATACGGTTCATCCGAATCAAAGTGCA |
| Q-WND6A-R |  |  |  | GTGAAATGAGGTCCGAACTCGAGCTA |
| Q-WND6B-F | Potri.019G083600 | | | GCCGTGCTCTCCAAGAACAGG |
| Q-WNDBA-R |  |  |  | GTGAAATGAGGTCCGAACTCGAGCTA |
| Q-PtrCCR2-F | Potri.003G181400 | | | GACCATTGTTGCAGCCCACT |
| Q-PtrCCR2-R |  |  |  | GGTAGGGATGGGGTACTCAG |
| Q-PtrCCOAOMT1-F | Potri.009G099800 | | | CATCTTTGTGGATGCTGACA |
| Q-PtrCCOAOMT1-R |  |  |  | GACGGCAGAGAGTGATGCCA |
| Q- PtrC4H2-F | Potri.019G130700 | | | AAGAGGTTCTGCACACGCAA |
| Q- PtrC4H2-R |  |  |  | AATCCCATGAGTTGCAGCCT |
| Q- PtrCOMT2-F | Potri.012G006400 | | | AACAGCCATTGAACTCGACC |
| Q- PtrCOMT2-R |  |  |  | GACACCATCCTCGTTCTTGGT |
| Q- PtrCAD1-F | Potri.009G095800 | | | ACTTGGAGGAGTAGGGCACA |
| Q- PtrCAD1R |  |  |  | AACAAACTGCAATGGGGCAT |
| Q- PtrF5H2-F | Potri.007G016400 | | | TTACAGAGCAGCTTTCGGGG |
| Q- PtrF5H2-R |  |  |  | GGCTAGCATGTCATCGACCA |
| Q- Ptr4CL5-F | Potri.003G188500 | | | AAATGGCGCAAATGGGGATG |
| Q- Ptr4CL5-R |  |  |  | GGGGCAGAGTCTACACACAT |
| Q- PtrCESA3A -F | Potri.002G257900 | | | CCAGGCAACCACTATGGAGAA |
| Q- PtrCESA3A -R |  |  |  | ATTAGGCTCACCCTCACGCT |
| Q- PtrGT43B -F | Potri.016G086400 | | | CCAGCTCCACCAAGCTCTAA |
| Q- PtrGT43B -R |  |  |  | ATGATCCAACTCTGCTTCGGG |
| Q- PtrGT8D -F | Potri.001G416800 | | | CTTGTCCAAAATGCGTTGCG |
| Q- PtrGT8D -R |  |  |  | GGCTTCTCGGTGTTATTTGCT |
| Q- PtrCESA2B -F | Potri.006G181900 | | | TGGAGCGGAGTAAGCATTGAG |
| Q- PtrCESA2B -R |  |  |  | TCCAGCAACAACTCCAACGA |
| **Vectors construction** | | | | |
| OE-MYB3-F | Potri.001G267300 | | | ATGAGGAAGCCGGATCTAATG |
| OE-MYB3-R |  |  |  | GTAGCCTGTTATAAAACTTGG |
| OE-WND6A-F | Potri.013G113100 | | | GTGGAGATGGAATCCTGTGTC |
| OE-WND6A-R |  |  |  | TGTTATATGTCAGGAAAGCAG |
| ProJAZ5-F(EcoR1)  1305 | Potri.006G139400 | | | CGGAATTCCTAGTCACTTGCTACAACGAC |
| ProJAZ5-R(BamH1)  1305 |  |  |  | CGGGATCCCTTCTGTAGTTTTCCAAATCTC |
| PtoJAZ5-OE-F  PtoJAZ5-OE-R  PtoJAZ5m-F  PtoJAZ5m-F  PtoJAZ5-1300-F  PtoJAZ5-1300-R | Potri.006G139400 | | | GCTCTAGAATGCCTTCAAGATCGTTATT  TCAAGTCTTGCCGGCAATTTTCC  ACAAGTTGTGGGTTGGCCACCC  GGGTGGCCAACCCACAACTTGT  ATGCCTTCAAGATCGTTATT  CTAACCAACTCTTACCCTATT |
| **BiFC** | | | | |
| PtoJAZ5-F（cYFP BamHI）  PtoJAZ5-R（cYFP XbaI）  WND6A-F（nYFP BamHI）  WND6A-R（nYFP XbaI）  MYB3-F（nYFP BamHI  MYB3-R（nYFP XbaI） | |  | tacaattacaggtacccggggatccGAAGATGTCTGGCT CGCTGCCACCGCCGTCGACTCTAGAGTGTGTTGTAGATGGAGC  caacatcgaggacgccggcggatccATGGAATCCTGTGTCCCACC  GAAAGCTCTGCAGGTCGACTCTAGATGTTATATGTCAGGAAAGCAG  caacatcgaggacgccggcggatccATGAGGAAGCCGGATCTAATG  GAAAGCTCTGCAGGTCGACTCTAGAGTAGCCTGTTATAAAACTTGG | |
| **PGBKT7** | | | | |
| JAZ1-F | Potri.001G062500 | | | CTGCATATGGCCATGGAGGCCGAATTCATGTCCAGAGCAGCAGCTGT |
| JAZ1-R |  |  |  | TGCGGCCGCTGCAGGTCGACGGATCCTTAGGTGTTGCAAACATACGG |
| JAZ2-F | Potri.001G166200 | | | CTGCATATGGCCATGGAGGCCGAATTCATGGCGAATTTAGCACAGAATTC |
| JAZ2-R |  |  |  | TGCGGCCGCTGCAGGTCGACGGATCCTTACAACTTAAGCTCTAGCTG |
| JAZ3-F | Potri.003G068900 | | | CTGCATATGGCCATGGAGGCCGAATTCATGGCGAATATGGCACAGAAATC |
| JAZ3-R |  |  |  | TGCGGCCGCTGCAGGTCGACGGATCCCTACAATTTAAGCTCGAGCTG |
| JAZ4-F | Potri.003G165000 | | | CTGCATATGGCCATGGAGGCCGAATTCATGAAAATGTCGAGAGGAAC |
| JAZ4-R |  |  |  | TGCGGCCGCTGCAGGTCGACGGATCCTCAAAAACGCGTCTTAGGTG |
| JAZ5-F | Potri.006G139400 | | | CTGCATATGGCCATGGAGGCCGAATTCATGTCTGGCTCGACGGAATTC |
| JAZ5-R |  |  |  | TGCGGCCGCTGCAGGTCGACGGATCCCTAGTGTGTTGTAGATGGAG |
| JAZ6-F | Potri.006G217200 | | | CTGCATATGGCCATGGAGGCCGAATTCATGACTTTTGTTGCCTCTGATC |
| JAZ6-R |  |  |  | TGCGGCCGCTGCAGGTCGACGGATCCTCAAGCAAGGTTGGAAGCAAC |
| JAZ7-F | Potri.008G133400 | | | CTGCATATGGCCATGGAGGCCGAATTCATGGAAAGAGATTTTTTGGG |
| JAZ7-R |  |  |  | TGCGGCCGCTGCAGGTCGACGGATCCTTAATAGGGTTCAGGGTTGG |
| JAZ8-F | Potri.010G108200 | | | CTGCATATGGCCATGGAGGCCGAATTCATGGAAAGAGATTTTTTGGG |
| JAZ8-R |  |  |  | TGCGGCCGCTGCAGGTCGACGGATCCTTAGTAGGGTTCGGGGTTGGC |
| JAZ9-F | Potri.011G083900 | | | CTGCATATGGCCATGGAGGCCGAATTCATGAAGAGAAACTGCAATCTCG |
| JAZ9-R |  |  |  | TGCGGCCGCTGCAGGTCGACGGATCCGTCTCGAGAGAAGATCAATGA |
| JAZ10-F | Potri.012G044900 | | | CTGCATATGGCCATGGAGGCCGAATTCATGGAGAGAGATTTCCTAGG |
| JAZ10-R |  |  |  | TGCGGCCGCTGCAGGTCGACGGATCCCTAGTTGCTGGCTGGGAGAGG |
| JAZ11-F | Potri.015G035800 | | | CTGCATATGGCCATGGAGGCCGAATTCATGGAGAGAGATTTTCTGGGTC |
| JAZ11-R |  |  |  | TGCGGCCGCTGCAGGTCGACGGATCCCTAATTGCTGGCTGGGAGAGGG |
| JAZ12-F | Potri.018G047100 | | | CTGCATATGGCCATGGAGGCCGAATTCATGGAGGCTCAACAACCTGATTC |
| JAZ12-R |  |  |  | TGCGGCCGCTGCAGGTCGACGGATCCTCAAACAACGTTGGCAGCAAC |
| **PGADT7** | | | | |
| MYB2-F | Potri.001G258700 | | | CATATGGCCATGGAGGCCAGTGAATTCATGAGGAAGCCAGAGGCCTC |
| MYB2-R |  |  |  | ATCTGCAGCTCGAGCTCGATGGATCCCTCAACTTTGGAAATCAAGAG |
| MYB3-F | Potri.001G267300 | | | CATATGGCCATGGAGGCCAGTGAATTCATGAGGAAGCCGGATCTAATG |
| MYB3-R |  |  |  | ATCTGCAGCTCGAGCTCGATGGATCCCTTATAAAACTTGGAAATCAAG |
| MYB20-F | Potri.009G061500 | | | CATATGGCCATGGAGGCCAGTGAATTCATGAGGAAGCCGGATCTAGTG |
| MYB20-R |  |  |  | ATCTGCAGCTCGAGCTCGATGGATCCCTTATTCTACGTGGAAATCAAG |
| MYB21-F | Potri.009G053900 | | | CATATGGCCATGGAGGCCAGTGAATTCATGAGGAAGCCAGAGGCCTC |
| MYB21-R |  |  |  | ATCTGCAGCTCGAGCTCGATGGATCCCTCATTGGAAATCAAGGAATG |
| MYB74-F | Potri.015G082700 | | | CATATGGCCATGGAGGCCAGTGAATTC ATGGGACGACATTCTTGTTG |
| MYB74-R |  |  |  | ATCTGCAGCTCGAGCTCGATGGATCCC GTTCATATCTGGTGGAAGAC |
| WND1A-F | Potri.011G153300 | | | CATATGGCCATGGAGGCCAGTGAATTC ATGCCTGAAGATATGGTG |
| WND1A-R |  |  |  | ATCTGCAGCTCGAGCTCGATGGATCCC TTGTTATACCGACAAGTGGC |
| WND1B-F | Potri.001G448400 | | | CATATGGCCATGGAGGCCAGTGAATTC ATGCCTGAGGATATGATGAATC |
| WND1B-R |  |  |  | ATCTGCAGCTCGAGCTCGATGGATCCC GTTATACCGATAAGTGGC |
| WND2A-F | Potri.014G104800 | | | CATATGGCCATGGAGGCCAGTGAATTC ATGACAGAAAACATGAGTAT |
| WND2A-R |  |  |  | ATCTGCAGCTCGAGCTCGATGGATCCC GTTATGCACCTGTGTTTGAC |
| WND2B-F | Potri.002G178700 | | | CATATGGCCATGGAGGCCAGTGAATTC ATGACAGAAAACATGAGTAT |
| WND2B-R |  |  |  | ATCTGCAGCTCGAGCTCGATGGATCCC GCTATACACTAGTGTTTGGC |
| WND3A-F | Potri.015G127400 | | | CATATGGCCATGGAGGCCAGTGAATTC ATGAATTCTTTTACACACGTTCC |
| WND3A-R |  |  |  | ATCTGCAGCTCGAGCTCGATGGATCCC GATATCATCACTTCCATAGATC |
| WND3B-F | Potri.012G126500 | | | CATATGGCCATGGAGGCCAGTGAATTC ATGAATACTTTTACACATG |
| WND3B-R |  |  |  | ATCTGCAGCTCGAGCTCGATGGATCCC ATATCATCATTTCCATAGATC |
| WND4A-F | Potri.001G120000 | | | CATATGGCCATGGAGGCCAGTGAATTC ATGAATACCTTCTCGCATGTC |
| WND4A-R |  |  |  | ATCTGCAGCTCGAGCTCGATGGATCCC TTCACTTCCATAGATCAATTTG |
| WND4B-F | Potri.003G113000 | | | CATATGGCCATGGAGGCCAGTGAATTC ATGAATACCTTTTCGCATG |
| WND4B-R |  |  |  | ATCTGCAGCTCGAGCTCGATGGATCCC AGCCTTCACTTCCACAGATC |
| WND5A-F | Potri.007G014400 | | | CATATGGCCATGGAGGCCAGTGAATTC ATGAAAAATCTTGACAAGC |
| WND5A-R |  |  |  | ATCTGCAGCTCGAGCTCGATGGATCCC GATTCATTTCTCAAATATGC |
| WND5B-F | Potri.005G116800 | | | CATATGGCCATGGAGGCCAGTGAATTC ATGAAAAAGCTCGACAAACAG |
| WND5B-R |  |  |  | ATCTGCAGCTCGAGCTCGATGGATCCC CTCTTCATTTTTCAAATATGC |
| WND6A-F | Potri.013G113100 | | | CATATGGCCATGGAGGCCAGTGAATTCATGGAATCCTGTGTCCCACC |
| WND6A-R |  |  |  | ATCTGCAGCTCGAGCTCGATGGATCCC TGTTATATGTCAGGAAAGCAG |
| WND6B-F | Potri.019G083600 | | | CATATGGCCATGGAGGCCAGTGAATTC ATGGAGATGGAATCCTGTGTC |
| WND6B-R |  |  |  | ATCTGCAGCTCGAGCTCGATGGATCCC GTTATTATAAGTCAGGAAAGC |
|  | | | | |
